# Influence of multi-species data on gene-disease associations in substance use disorder using random walk with restart models

**DOI:** 10.1371/journal.pone.0325201

**Published:** 2025-06-16

**Authors:** Everest U. Castaneda, Sharon Moore, Jason A. Bubier, Stephen K. Grady, Michael A. Langston, Elissa J. Chesler, Erich J. Baker

**Affiliations:** 1 Department of Biology, Baylor University, Waco, Texas, United States of America; 2 School of Engineering and Computer Science, Baylor University, Waco, Texas, United States of America; 3 The Jackson Laboratory, Bar Harbor, Maine, United States of America; 4 Department of Electrical Engineering and Computer Science, University of Tennessee, Knoxville, Tennessee, United States of America; 5 Department of Mathematics and Computer Science, Belmont University, Nashville, Tennessee, United States of America; Chinese Academy of Sciences, CHINA

## Abstract

A major challenge lies in discovering, emphasizing, and characterizing human gene-disease and gene-gene associations. The limitations of data on the role of human gene products in substance use disorder (SUD) makes it challenging to transition from genetic associations to actionable insights. The integration of data from multiple diverse sources, including information-dense studies in model organisms, has the potential to address this gap. We demonstrate a modified performance of the Random Walk with Restart algorithm when multi-species data is integrated in the heterogeneous network within the context of SUD. Additionally, our approach distinguishes among disparate pathways derived from the Kyoto Encyclopedia of Genes and Genomes. Thus, we conclude that direct incorporation of multi-species data to an aggregated heterogeneous knowledge graph can adjust RWR’s performance and enables users to discover new gene-disease and gene-gene associations.

## Introduction

Emphasizing gene-disease relationships out of global genomic data resources can lead to the discovery of novel preventative treatments or pharmaceutical targets for complex trait disorders [1, 2]. A wealth of genome-wide experimental techniques can rapidly generate data across many populations, species, and study types, providing holistic data resources for the identification of biological mechanisms of disease [3]. Data from proteomic, genomic, and metabolomic studies are often composed into multi-omics data structures for further study to mitigate issues related to data incompleteness and unevenness [4], which are due to limitations on experimental techniques, fluctuations of specific research interests, and variation in the application and nature of conservation in model organisms [5]. Multi-omics graphs are built from independent experiments, such as genome-wide association studies (GWAS), differential expression studies, and drug-interaction studies, among others [6]. As an integral aspect of a multi-omics graph, GWAS studies provide valuable links between human genetic variants and complex disease traits in diabetes [7], substance use disorders (SUD) [2], and cancer [8].

A major goal of genome wide genetic analysis is to find genes and their associated actionable biological mechanisms to facilitate prevention, diagnostics, and therapeutics [9]. Human genetic studies face challenges due to the limited breadth, environmental control, and ability to profile intrinsic biological mechanisms [9]. In SUD research, the heterogeneity and complexity of drug exposure history limits the power of human genetic studies, and in studies of rare disease few cases exist [10] from which to form the associations, leading to challenges with data incompleteness and unevenness when attempting to infer gene-disease relations [11, 12]. The wealth of data available across multiple species can be used to develop gene-gene and gene-disease associations, filling in areas of incomplete data in humans and bridging the gap between complex disease and simpler biobehavioral processes that are readily characterized in model organisms. Psychiatric disorders prove to be a particularly interesting application area because their underlying genetic etiology [13, 14] exists on a continuum [15], which operate trans-diagnostically across multiple disorders and encompass a variety of phenotypes [16, 17]. Despite the high heritability of these conditions, their complexity often makes precise characterization, classification, diagnosis, and therapeutic target discovery quite challenging [2]. Data around these disorders is uneven, particularly as more specific subsets of conditions are considered [2, 18]. In human genetic analysis of SUDs, alcohol use disorder has seen a large increase in power, but other disorders, such as cocaine use disorder and opioid use disorder, have not [19–21].

SUDs are studied using a variety of approaches ranging from human GWAS studies to specific gene-centric neurobiological investigation often executed in model organisms [22]. One approach to increase statistical power is to aggregate positive associations from GWAS studies and pool data related to each disease [23–26]. Most approaches limit the scope of data integration to a single species, which when taken in context of the incompleteness and unevenness of human data, limits the breadth of results obtained. In contrast, data from animal populations provide depth and breadth of gene associations related to SUDs and the many neurobiological and behavioral processes that confer disease vulnerability [19]. The limitations imposed by data incompleteness and unevenness in human integrative studies may be alleviated by the incorporation of data from multiple species in which the neurobiological and genomic underpinnings of various aspects of the disease can be more comprehensively characterized [22]. To take advantage of this data, computational methods that perform well against a backdrop of uneven, biased, or missing data [27], are needed to harmonize and utilize existing animal model and human data at all phenotypic levels [19].

The relationships among genes, biological functions, and disease processes are often enumerated and cataloged in biological databases as individual assertions in data tables and relational databases, but are best represented in one-to-many or many-to-many networks [28]. Contemporary analysis methods can capture novel gene-gene and gene-disease associations in networks derived from homogeneous data sources [29] or heterogeneous networks [27, 30–32]. A heterogeneous network is a multi-omics biological data representation in which protein-protein interactions, gene-disease associations, gene-ontology representations, and other biological association data are coalesced to form a contiguous, biologically-relevant knowledge map [5, 30]. The application of graph algorithms has enabled the interrogation of the interactome underlying these networks [33] with varying approaches including diffusion metrics based on the random walk algorithm [30, 34, 35], connectivity significance [36, 37], and Markov clustering [38], among others [39].

Previous studies have demonstrated the utility of the random walk with restart (RWR) algorithm to explore the relatedness of biological entities within a heterogeneous graph [30, 40–43]. However, these prior studies have largely been species specific [44], and may benefit from taking advantage of the vast reserve of information on homologous gene products in multi-species data. Here, we evaluate the utility of directly incorporating aggregated and experimentally derived model organism data across species into the heterogeneous graph.

We present an analysis of multi-species data within the context of complex psychiatric disorder knowledge maps derived from genomic studies. By using multi-species networks in conjunction with RWR we demonstrate a discovery approach, and we compare the results of recapitulating genes involved in multiple SUDs using multiple species versus single species and also compare RWR to a module-based gene-disease approach, Disease Module Detection (DIAMOnD) [36]. Furthermore, we illustrate the extrapolation of a multi-species SUD-centric graph to logically associate gene-gene relationships in functionally distinct metabolic pathways and compare it to semantic similarity scoring [45].

## Materials and methods

### Heterogeneous network assembly

**Fig 1:**
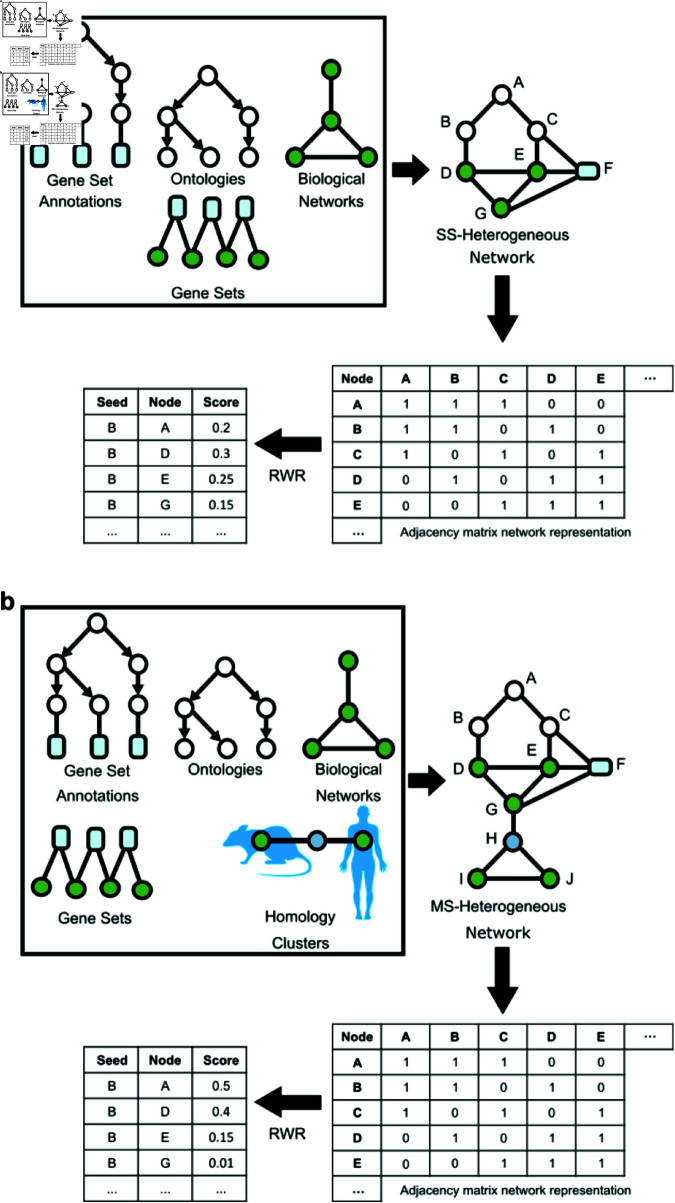
Scheme for building heterogeneous graphs and subsequent RWR. We outline the creation of two graphs used in this analysis as well as the generalized data sources and the RWR process with artificial walk scores. Gene sets were acquired from GeneWeaver along with their subsequent ontologies. Biological networks were acquired from Biological General Repository for Interaction Datasets and the Kyoto Encylopedia of Genes and Genomes. We directly integrate the Gene Ontology Biological Process map. For the multi-species graph, we incorporated homology clusters and aggregated data to include biological networks and gene sets from *Homo sapiens*, *Mus musculus*, and *Rattus norevigicus*. (a) Graph building and subsequent graph walk for the single species graph. (b) Multi-species graph building and graph walk. Note that the inclusion of the homology clusters is highly contrasted to the single species approach, which allowed input for additional networks derived from the 3 species.

In our analysis, we used an implementation of an RWR using NetworkX [46], Pandas [47], and SciPy [48] to aggregate all graphs, which is available at (https://github.com/treynr/ness). We constructed biological graphs for multi-species and single species heterogeneous networks, which are both unweighted and undirected graphs. All human and animal data was acquired from publicly available repositories, which completely anonymize and follow all regulations prior to public dissemination, hence there are no ethical concerns. The single species graph, Fig 1a, was formed from publicly available data, which includes genomic interactions, ontologies, genesets, and annotations. The multi-species graph, Fig 1b, was formed in a similar manner but with multi-species data alongside homological clusters [49] derived from the Alliance of Genome Resources (AGR) [50]. Briefly, a heterogeneous, multi-species biological network is constructed from distinct publicly available repositories. The resulting agglomerate graph’s biological entities’ affinities are measured via an RWR. The results are given as an edge list of source and target dynamics with probability scores determining the direct relationship between two entities. The three species we used in this study were *Homo sapiens*, *Mus musculus*, and *Rattus norvegicus*. All data were sourced from publicly available repositories, including the following: Gene Ontology Resource (GO) [51], The Biological General Repository for Interaction Datasets (BioGrid) [52], Alliance of Genome Resources (AGR) [50], Kyoto Encyclopedia of Genes and Genomes (KEGG) [53], and GeneWeaver (GW) [54]. GW (https://geneweaver.org/) is a suite of services, which contains data, tools, and resources for integrative genomics analyses [54]. GW uses a bipartite model projection, which combines publicly sourced and expertly curated gene associations into a single numerical identifier, termed Gene Set IDs (GSIDs). For this study we used GSIDs consistent with nicotine use disorder, alcohol use disorder, heroin use disorder, and morphine use disorder. Homology data was featured from two sources, GW homology clusters and AGR [49, 50]. GW features homology clusters from NCBI Homologene [55] and Mouse Genome Informatics [56]. We gathered homologous genes for each of the three species in the AGR database [56], creating a homology edge list. For AGR derived data, a count of ≥9 was used for our orthology inclusion threshold. Briefly, counts are derived from distinct algorithms housed in different database repositories such as InParanoid [57], Ensembl Compara [58], and PANTHER [59]. Ontology data was sourced from the Gene Ontology Resource [51, 60]. All genetic data from public repositories were converted into NCBI gene IDs [61], where available. Genes which had no NCBI gene ID were mapped to UniProt KnowledgeBase IDs [62].

### Protein-protein interaction network assembly

Protein-protein interaction (ppi) data was gathered from KEGG [53] and BioGRID [52]. KEGG pathways and BioGRID interactions were sourced from the 3 species of interest, *Mus musculus*, *Homo sapiens*, and *Rattus norvegicus*. For KEGG pathway graphs, we focused on “Metabolism”, “Genetic Information Processing”, “Environment Information Processing”, “Cellular Processes”, and “Organismal Systems” for inclusion since these pathways typically contain gene networks. We excluded “Human Disease” and “Drug Development” due to the species-exclusive nature of the former and the latter containing scant usable gene-gene associations. KEGG pathway networks used in both multi-species and single species graph were parsed using the KEGG NetworkX Topological Parser [63] with the *knext genes* command. Furthermore, for the global single species ppi graph, we only used human versions of the two datasets noted.

### Gene ontology

Ancestors and descendants that were “is a”, “part of”, and “regulates” in association were gathered from the biological process root to form our ontology graph. We excluded electronically inferred annotations from the ontology graph, and hence, we only sourced ontology annotations that were verified through experimentation or expertly curated sources, which only included the following evidence codes: EXP, IDA, IEP, IGI, IMP, IPI, TAS, IC. These ontologies were also used in conjunction with all genes in our heterogeneous and interaction graphs to create a gene-ontology graph. We define the closure of a node as the total number of genes associated with that Biological Process terms and genes associated with any “is a”, “part of,” or “regulates” child term added recursively to each leaf biological process term. We only included the closure of all annotation sets such that every ancestor term is annotated to any gene that is annotated to any of its descendants for nodes that contained ≤200 genes, see Supporting information S1 Fig. This threshold was set due to the generalization of upper level ontology terms [64], thereby reducing the number of edges analyzed by RWR, which has a complexity of $\text{O}({\text{n}}^{2})$.

### Comparison of SUD-associated genes

For comparisons of DIAMOnD and RWR, genes related to alcohol, morphine, heroin, and nicotine use were acquired from DisGeNET [65], which is an expertly curated database of gene-disease associations. Genes known to produce a particular phenotype have been used as a basis for cross validation studies in previously published works [30, 66]. Here, we used DisGeNET [65] as our reference and randomly split genes from the database in a 5-fold cross-validation study. Training sets were used to seed the algorithms, and we determined the proportion of recapitulated genes based on how many test set genes were given as output and divided this by the total amount of genes in the test set.

### Comparison of KEGG genes

KEGG was used for our comparison of the discriminative property of an assortment of distinct metabolic pathways, given semantic similarity comparisons to RWR. KEGG has been leveraged in several previous studies for benchmarking semantic algorithms. 13 distinct pathways, used in previous studies as representative, highly diverse human pathways, were used in this study, which are listed in Table 1.

**Table 1:** KEGG pathway used in discriminative power benchmarking experiments.

Pathway Code	Pathway Name	Total
hsa:03430	Mismatch Repair	23
hsa:03020	RNA polymerase	34
hsa:03022	Basal transcription factors	45
hsa:00670	One carbon pool by folate	20
hsa:00920	Sulfur metabolism	10
hsa:04950	Maturity onset diabetes of the young	25
hsa:00563	Glycosylphosphatidylinositol (GPI)-anchor biosynthesis	26
hsa:03450	Non-homologous end-joining	13
hsa:00232	Caffeine metabolism	6
hsa:04130	SNARE interactions in vesicular transport	33
hsa:00140	Steroid hormone biosynthesis	62
hsa00290	Valine, leucine and isoleucine biosynthesis	4
hsa:00040	Pentose and glucuronate interconversions	36

Total reflects the total amount of genes in the pathway.

### Random walk with restart

In this work, we denote vectors using bold lower-case letters. Subscripts denote the start node for the vector. The *i*-th entry of the vector **p** with start node **u** is denoted as $p_{u}(i)$. RWR [30, 34, 67] was used to gauge the distance between two nodes in a graph. In previous studies, the RWR algorithm was shown to be more effective than clustering and neighborhood approaches in predicting gene-disease associations or prioritization in both protein-protein interactions and heterogeneous graphs [30, 39]. The RWR algorithm begins at a user-defined seed node and then produces a probability distribution of visiting a node from the initial seed, and a restart probability can be user defined [30, 67]. A high restart probability focuses on the local topology while a lower restart probability focuses globally [30]. Briefly, the probability of visiting a node in a graph can be defined as Eq 1.

$$p_{u}^{\left(t+1\right)}=(1-r)Ap_{u}^{t}+re_{u}\text{ , }\;p_{u}^{\left(0\right)}=p_{s}$$ (1)

where *A* is the column-normalized adjacency matrix, $p_{u}^{t}$ is the proximity vector of node *u* at iteration *t*, $p_{s}$ is the “user-defined” start vector, $e_{u}$ is the seed unit vector, and r∈(0,1) is the restart probability. The seed unit vector, $e_{u}$, is defined as $e_{u}(i)=1$ when i=start and in the case of a single start node $e_{u}(j)=0$ for i≠j, 1≤i≤n, but in the case of multiple starts $e_{u}(j)=1$ for i≠j, 1≤i≤n. $p_{u}$ is calculated iteratively until a steady state is reached. Furthermore, we used a restart probability of 0.25 for all analyses, which has been shown to be effective in other studies [30]. The algorithm stops once the *L*_1_ norm of the difference between proximity vectors at the two successive iterations reaches a sufficiently low value, defined as τ, see Eq 2. Here we used a threshold of $\tau =10^{-8}$.

$$∥p^{\left(t+1\right)}-p^{t}{∥}_{1}<\tau$$ (2)

### Semantic similarity metric

For the comparison between RWR and baseline semantic similarity measures, we begin by using known gene-gene interactions in the form of KEGG pathways. KEGG pathways are highly curated and offer a standard in which to determine the effectiveness of both measures. We overlaid functional similarity Eq 3 to the genes involved in a KEGG pathway [68]. This methodology employs the maximum semantic similarity from the annotation of both sets of genes.

$$Sim(g_{ki},g_{kj})=\frac{\sum_{t\in T_{1}}Sim_{terms}(t,T_{2})+\sum_{t\in T_{2}}Sim_{terms}(t,T_{1})}{\left|T_{1}|+|T_{2}\right|}$$ (3)

Summarily, *T*_1_ and *T*_2_ are terms annotated to genes, *g*_*ki*_ and *g*_*kj*_, in some pathway, *k*, and the semantic similarity score, *Sim*_*sem*_, is defined as Eq 4. *Sim*_*sem*_ can be any semantic similarity measure.

$$Sim_{terms}(t,T)=\max_{t^{\prime }\in T}Sim_{sem}(t,t^{\prime })$$ (4)

In this study, *Sim*_*sem*_ is replaced with Lin [69, 70], Jaccard [70, 71], Resnik [72], and cosine [70] measures. Lin and Resnik similarity measures have historically been utilized for benchmarking of KEGG information retrieval [45, 68, 73, 74] while Jaccard and cosine measures have been used in ontological semantic similarity analyses [70, 75]. For comparisons of semantic similarity metrics to RWR, we leveraged the discriminating power [73]. This metric follows that pathways which share genes should have high intraset similarity and high discriminatory power due to their similar biological processes, Eq 5 [73, 74]. This measure evaluates an algorithm’s ability to differentiate between two or more functionally distinct metabolic pathways [73]. Briefly, $S=\{S_{1},S_{2},...,S_{i}\}$ is some collection of KEGG pathway of *b* genes. For this study, *p* is the size of the collection of *S* that does not contain *S*_*k*_. $Sim(g_{ki},g_{kj}$) is some similarity measure, in our case the measures stated above, and *b* is the set of genes that make up pathway *S*_*k*_. Finally, *InterSetSim* is the measure of similarity between two pathways, *S*_*k*_ and *S*_*l*_, composed of *b* and *c* genes.


$$IntraSetSim(S_{k})=\frac{\sum_{i=1}^{b}\sum_{j=1}^{b}Sim(g_{ki},g_{kj})}{b^{2}}$$

$$InterSetSim(S_{k},S_{l})=\frac{\sum_{i=1}^{b}\sum_{j=1}^{c}Sim(g_{ki},g_{lj})}{b\times c}$$

$$DP_{sim}(S_{k})=\frac{\left(p-1)IntraSetSim(S_{k}\right)}{\sum_{i=1,i\neq k}^{p}InterSetSim(S_{k},S_{i})}$$ (5)

### DIAMOnD implementation

For the comparisons with DIAMOnD, we leveraged both human interaction data and homologous graph data. Briefly, DIAMOnD is an iterative, parameter-free algorithm that determines the connectivity significance of disease associated proteins [36]. With a list of user-defined seed genes, DIAMOnD grows a disease module through calculating the seeds module’s connectivity significance with other genes. DIAMOnD defaults to return 200 DIAMOnD genes as this results in a subgraph with topological properties that mimic real disease with interaction rates higher than random chance [36]. For our purposes, we returned the first 250 nodes to make sure to recapitulate as many test genes as possible, which is still within the recommended ~200 range [36].

### Two-fold filter

RWR ranks nodal associations based on their relative affinities, but we also established the significance of these affinities through graph permutations. We added a two-fold threshold for genes that were significant in our comparison to DIAMOnD. Our first threshold was probability, after permutation testing, ≤0.01. Briefly, permutation testing involved randomly rearranging all nodes in the graph and repeating the random walk. A p-value was derived from observing a probability as high or higher than the initial observation. We conducted 1000 permutation tests for all trials. Furthermore, we filtered out all genes which had a walk probability lower than the 95th quantile of walk all scores.

### Statistical comparisons

Statistical tests were conducted for cross comparison of performance for DIAMOnD, semantic algorithms, ms RWR, and ss RWR. A Mann-Whitney U test was conducted for comparison of the proportion of recapitulated genes, which involved the comparison between DIAMOnD, ss RWR, and ms RWR. For the Discriminative Property comparison, a Kruskal-Wallis Analysis of Variance was performed to test statistical significance between semantic and RWR scoring. Dunn’s post-hoc tests were corrected for multiple comparisons using a Benjamini-Hochberg correction, see Supporting information S1 Table. Furthermore, we used Cliff’s Delta (δ) for all pairwise comparisons to determine the effect-size [76]. We used the *effsize* package [77] in R version 4.3.1 [78] with default parameters, which included the following: a confidence level of 0.95, “unbiased” estimate, and a Student-t distribution. Interpretation of effect size was from (Hess and Kromrey, 2004) [79] where δ<0.147 is a small effect, 0.147<δ<0.330 is a moderate effect, and a large effect is δ>0.474 [80].

## Results

### Comparison of RWR to a contemporary method to recapitulate disease genes

Our results of benchmarking analyses using RWR in heterogeneous biological networks are derived from single species data sets (ss RWR) and multi-species data sets leveraging clusters of homologous genes (ms RWR). Our initial comparisons were to determine the enhancement compared to single species approaches in recapitulating genes involved in multiple SUDs. We used the proportion of recapitulated genes as a measure of improvement between the different graph types and algorithms. The more genes known to be associated with SUDs recapitulated offers insight into the performance.

### Analysis of RWR and DIAMOnD in recapitulating genes involved in multiple substance use disorders

**Fig 2:**
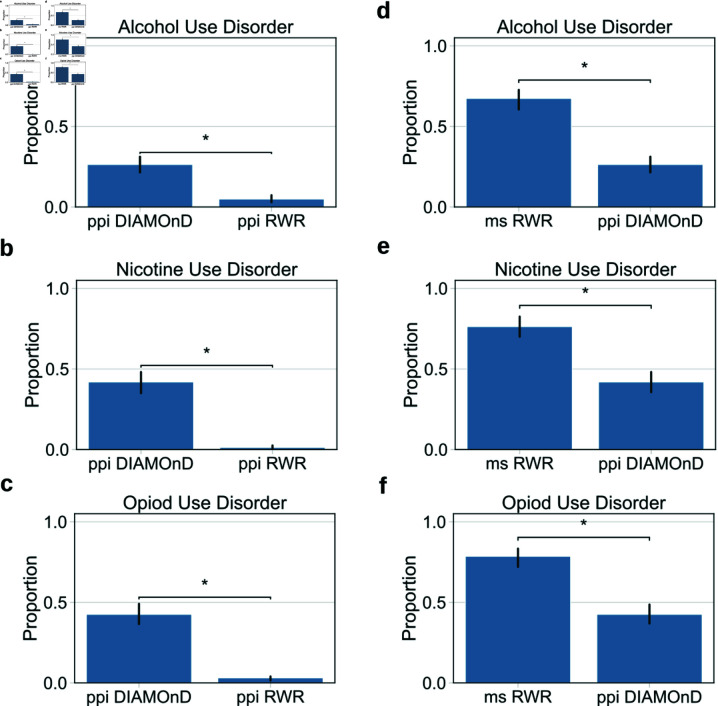
Statistical comparisons of the proportion of recapitulated genes for three substance use disorders. Statistical comparison was made using a Mann-Whitney U test. Comparisons with DIAMOnD were made against either a global single species protein-protein interaction (ppi) graph, a-c, or a multiple species heterogeneous graph, d-f. (a) DIAMOnD’s proportion of recapitulated genes was significantly higher than RWR for the alcohol use disorder (AUD), $U-statistic=2.50\;\times \;10^{1}$ and $p-value=1.12\;\times \;10^{-2}$. (b) DIAMOnD’s proportion of recapitulated genes was significantly higher than RWR for the nicotine use disorder (NUD), $U-statistic=2.50\;\times \;10^{1}$ and $p-value=1.07\;\times \;10^{-2}$. (c) DIAMOnD’s proportion of recapitulated genes was significantly higher than RWR for opioid use disorder (OUD), $U-statistic=2.50\;\times \;10^{1}$ and $p-value=1.19\;\times \;10^{-2}$. (d) RWR was significantly higher for the multi-species (ms) AUD graph, $U-statistics=2.50\;\times \;10^{1}$ and $p-value=1.14\;\times \;10^{-2}$ (e) RWR was significantly higher for the NUD ms graph, $U-statistics=2.50\;\times \;10^{1}$ and $p-value=1.14\;\times \;10^{-2}$ (f) RWR was significantly higher for the OUD ms graph, $U-statistics=2.50\;\times \;10^{1}$ and $p-value=1.22\;\times \;10^{-2}$.

We conducted a comparison of RWR in single or multiple species against a modular approach, DIAMOnD [36]. Our benchmark for comparison was determined as the capability to recapitulate genes known to have an association to a group of SUDs: alcohol use disorder (AUD), nicotine use disorder (NUD), and opioid use disorder (OUD). For the first comparison, we used global single species interaction data that is optimized for DIAMOnD’s single species modular approach [36]. In this case, DIAMOnD outperformed RWR through verifying significance using a Mann-Whitney U test in AUD ($U-statistic=2.50\;\times \;10^{1}$ and $p-value=1.12\;\times \;10^{-2}$), NUD ($U-statistic=2.50\;\times \;10^{1}$ and $p-value=1.07\;\times \;10^{-2}$), and OUD ($U-statistic=2.50\;\times \;10^{1}$ and $p-value=1.19\;\times \;10^{-2}$), see Fig 2a–2c, which is similar to results cited by Ghiassian *et al*. [36]. While this was true for the ppi network, for the multi-species graph, RWR outperformed DIAMOnD, which failed to recapitulate any disease genes. We then conducted a cross-comparison, Mann-Whitney U test, of the two best performing comparisons. Multi-species RWR outperformed ppi DIAMOnD in recapitulating genes associated with AUD ($U-statistic=2.50\;\times \;10^{1}$ and $p-value=1.14\;\times \;10^{-2}$), NUD ($U-statistic=2.50\;\times \;10^{1}$ and $p-value=1.14\;\times \;10^{-2}$), and OUD ($U-statistic=2.50\;\times \;10^{1}$ and $p-value=1.22\;\times \;10^{-2}$), see Fig 2d–2f. The results show a significant improvement in the RWR model when multi-species data is added.

### Comparative analysis of using ontologies in recapitulating KEGG’s discriminative property

**Fig 3:**
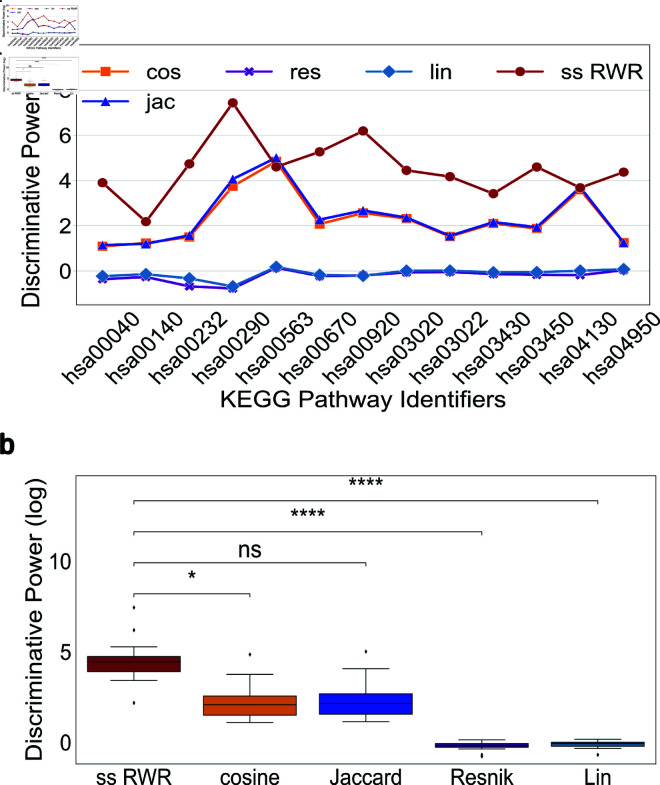
Comparison of RWR against semantic similarity measures for single species data. Probability value (*p*) annotation are as follows: ns: $5.00\;\times \;10^{-2}<p\leq 1.00$, *: $1.00\;\times \;10^{-2}<p\leq 5\;\times \;10^{-2}$,**: $1.00\;\times \;10^{-3}<p\leq 1.00\;\times \;10^{-2}$ ***: $1.00\;\times \;10^{-4}<p\leq 1.00\;\times \;10^{-3}$ ****: $p\leq 1.00\;\times \;10^{-4}$ (a) Single species RWR (ss RWR) outperformed all other semantic similarity measures in 11 of 13 pathways. Jaccard outperformed ss RWR in 2 pathways while cosine outperformed ss RWR in 1 pathway. (b) Dunn’s test comparison of ss RWR with all semantic similarity measures. discriminative power was significantly higher than all other algorithms except Jaccard.

Within the context of SUDs, an accurate representation of biological pathway information can offer insights to the relevance of proteins and genes within our approach [81] and if this insight is enhanced with multiple species data. Hence, we tested the recapitulation of a cohesive foundation of reliably observed molecular interactions [45, 82]. If we can recapitulate consistent interactions and suppress spurious interactions, we can increase confidence in the novel associations extracted from ms RWR within the context of SUDs. We compared the relations identified using each approach, ss RWR and ms RWR, to those extracted using contemporary semantic similarity techniques. Hence, for the semantic similarity approaches, we relied on GO Biological Process annotations as a baseline background dataset to estimate gene-gene functional similarity with the assumption that genes in the same pathway will oftentimes share biological processes [73]. Here, we compared ss RWR and ms RWR to Resnik [72], Lin [69], cosine [70], and Jaccard [71]. We performed a comparison of discriminative power (DP), a measure of the functional cohesiveness of a set of metabolically distinct genes [73], for single species and multi-species knowledge graphs. From our comparison of RWR to traditional semantic similarity measures, we found that ss RWR outperformed baseline semantic measures in 11 of the 13 pathways, see Fig 3a. Furthermore, DP was significantly different, $H-statistic=5.20\;\times \;10^{1}$ and $p-value=1.41\;\times \;10^{-10}$, in ss RWR versus all other measures using a Kruskal-Wallis Analysis of Variance (KW-ANOVA). In pairwise Dunnett’s post-hoc comparison tests, ss RWR outperformed cosine ($p-value=4.54\;\times \;10^{-2}$), Resnik ($p-value=2.31\;\times \;10^{-8}$), and Lin ($p-value=1.62\;\times \;10^{-7}$), but it did not significantly outperform Jaccard ($p-value=6.24\;\times \;10^{-2}$), see Fig 3b for more details. Moreover, ss RWR was robust to noise and missing data, see Supporting information S2 Fig–S4 Fig.

**Fig 4:**
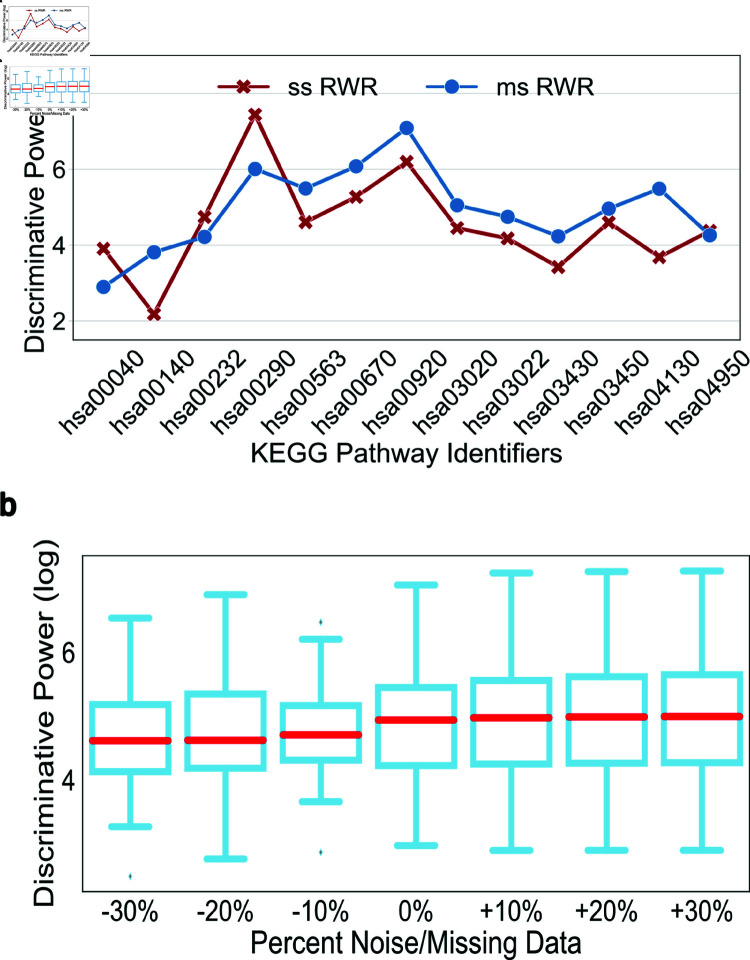
Comparison of RWR coupled with single species and multi-species data sources. (a) Multi-species (ms RWR) outperformed single species (ss RWR) in all pathways except the following 4 pathways: hsa00040, hsa00232, hsa00290, hsa04950. (b) Robustness of ms RWR to noise and missing data. KW ANOVA results show no significance between the discriminative property of any of the graphs, H−statistic=1.23 and $p-value=9.75\;\times \;10^{1}$.

Next, we conducted a direct comparison to evaluate the performance of ss RWR and ms RWR. ms RWR outperformed ss RWR in 9 of the 13 KEGG pathways, see Fig 4a. While ms RWR proved highly effective, it was also robust to random noise and missing edges up to 30% upon KW-ANOVA comparison, H−statistic=1.23 and $p-value=9.75\;\times \;10^{1}$, see Fig 4b, Supporting information S5 Figa–S5 Figc, and Supporting information S6 Figa–S6 Figc. Furthermore, in contrast to ss RWR, ms RWR outperformed all semantic similarity comparisons in all KEGG pathways Supporting information S7 Fig and S8 Fig. In pairwise Dunnett’s post-hoc comparison tests, ms RWR outperformed cosine ($p-value=2.11\;\times \;10^{-2}$), Resnik ($p-value=6.76\;\times \;10^{-9}$), Lin ($p-value=5.16\;\times \;10^{-8}$), and Jaccard ($p-value=2.97\;\times \;10^{-2}$), see Supporting information S7 Figb.

### Effect size comparisons

**Fig 5:**
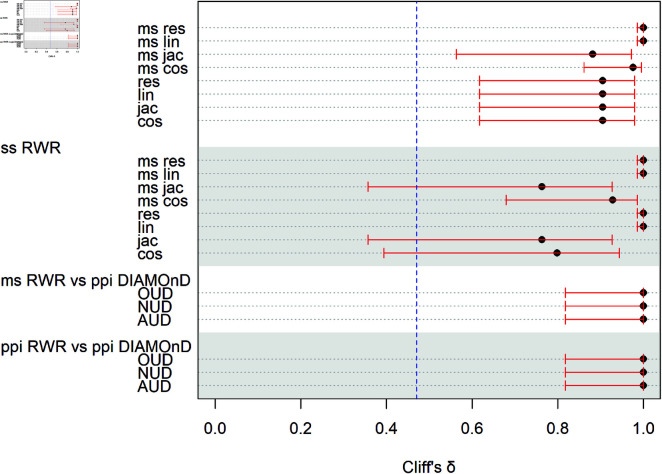
Cliff’s delta effect size for all comparisons. Blue vertical line shows where the threshold between a large and medium effect lies, δ<0.47. There was a large effect, within the 0.95 confidence interval, for all comparisons of ppi DIAMOnD and ppi RWR and all comparisons of ms RWR and ppi DIAMOnD. Comparisons of ss RWR and all semantic scoring all had a large effect, but the confidence interval went below threshold for ss RWR comparisons with ms jac, jac, and cos. No comparisons for ms RWR went below threshold.

We conducted a Cliff’s δ effect size to determine a standardized difference in significance. All comparisons between SUD had a large effect, see Fig 5. Moreover, most comparisons’ confidence intervals were above the “large effect” threshold, δ<0.47. Only comparisons between ss RWR and three semantic scorings, which included the following: multi-species Jaccard, single species Jaccard, and single species cosine, ran below threshold, see Fig 5.

## Discussion

In this study we present a framework for extraction of gene-disease associations using multi-species heterogeneous graphs and perform a comparative evaluation with other contemporary methods. Our results illustrate the addition of multi-species data modified RWR’s capabilities and outperformed a modular-based technique in recapitulating disease genes due to the inclusion of data on homologous gene products. The aggregated data sets used here provide unique insights not obtainable from single species data alone. Our study reinforces the need for model organism data reuse and integration as it provided enhanced results.

A challenge in working with diverse data is the heterogeneity of data structures involved. These datasets include bipartite representation of gene sets from GeneWeaver [54], undirected interactions from BioGrid [52], and annotations to a structured vocabulary like Gene Ontology (GO) Biological Process (BP) [51]. The use of the GO BP map has similar elements to the gene-ontology edge list used in Gentili *et al*. [30], but in contrast, we directly integrated the GO BP map into our heterogeneous network. The cut-off of the GO tree is due to the high generalizations of information that exist at the foundational levels [64]. While a full analysis of all GO combinations at all levels is beyond the scope of this article, we used a cut-off which allowed for whole graph permutations on our architecture. Hence, we compensated for this simplification with the copious information added by homology, but this careful trade-off revealed a direct increase in performance of RWR. We thereby demonstrate the richness of information offered by leveraging multiple species data through gene orthology, and these data are often overlooked in prior studies.

We noted a significantly high discriminative power [73, 74] compared to all semantic scoring of biological pathway-associated genes with our multi-species knowledge map indicating the underlying association data has a staunch metabolic foundation. Furthermore, we noted the robustness of the RWR algorithm to added noise and missing edges since both ss RWR and ms RWR were not significantly affected. By extension, we demonstrate the results support the strong interplay between metabolic pathways and addiction [83, 84]. In contrast to RWR, the added multi-species data significantly added more information, see Supporting information S9 Fig, but proved to worsen KEGG pathway recapitulation with semantic algorithms, see Supporting information S10 Fig. This reveals a vulnerability in semantic algorithms to filling in missing values with homological information as this increases infrequently observed relations, which might be a direct consequence of the unevenness and incompleteness of coverage in the GO tree [85, 86]. Moreover, genes embedded in both the ss RWR and ms RWR contain elements of KEGG pathways [87], which may introduce bias compared to semantic scoring, which relies solely on GO. More experiments will be needed to conduct a thorough investigation of the potential for bias in direct comparisons between semantic scoring and other guilt-by-association algorithms. Furthermore, DIAMOnD was incapable of recapitulating disease genes from our homology data map. As a proxy for this direct comparison, we compared DIAMOnD ppi to ms RWR because we were unable to recapitulate disease genes with ms DIAMOnD. While typically guilt by association algorithms perform better with more information [88], the dependency between DIAMOnD and RWR is most likely due to the optimization of DIAMOnD for local information and RWR for global information [30, 89, 90]. Even though PPI data is embedded in our heterogeneous network, the local agglomerative process by DIAMOnD may not overcome the noise generated by non-PPI relevant nodes [90, 91]. A more thorough comparison would attempt to rectify and balance both global and local information [89] as ppi DIAMOnD outperformed the ppi walk approach, but a comparison of this magnitude is beyond the scope of this article. Collectively, these considerations indicated that a successful algorithm leveraging multi-species data must be robust to noise, information distance, and missing edges.

Several other metrics exist for estimating semantic similarity [73, 74, 92] and clustering [93, 94], and although our analysis performed favorably on a comparison of well-known, well-documented baseline metrics that have been employed in previous studies [30, 70], it is possible that other algorithms may provide different results. The two pathways, hsa00232 and hsa00290, in which ms RWR performed poorly compared to ss RWR could be attributed to the modest number of genes, 6 and 4 genes in total respectively. This potentially reflects a certain threshold whereby single species graphs might perform better. However, a possible explanation might be due to the sheer number of edges, which is hinted in ms RWR’s increase in retrieving information from the aforementioned pathways when removing up to 30% of total edges, see Supporting information S6 Fig.

KEGG’s database of pathways are highly supported biological systems maps [73, 74], but they have issues with uncertainty due to inconsistent interactions and missing genes [95–97]. This uncertainty includes the absence of an event, ambiguous definitions of nodes and compounds [95], and the “missing gene” problem whereby target pathways do not have an equivalent gene in its orthologous template pathway [53, 98]. Proposals to bridge the gaps of uncertainty have included sequence homology and probabilistic likelihood for prioritized pathway filling [99–101], but approaches to gap-filling have limitations due to the uncertainty of the underlying databases [63, 102–104]. With the fundamental trade-off in consistency versus sparsity outlined in metabolic pathway recapitulation, we have chosen KEGG as our proxy to demonstrate our framework’s effectiveness against otherwise sparse and noisy data sets. We propose future experiments to leverage our multi-species framework to fill in missing pathway data within the context of SUDs. Furthermore, we stress the derived probability scores from our multi-species approach alongside RWR may provide coverage for an entire knowledge map and be used as edge weights to be combined with other algorithms such as shortest path, minimum spanning tree, and maximal flow. We hope to apply our multi-species framework in future studies to emphasize poorly studied genes in complex traits [105] or discover novel gene-gene relationships.

The ms RWR’s framework is shown to be effective in identifying genomic associates of psychiatric conditions. Our findings corroborate previous analyses that show a RWR approach coupled with heterogeneous data outperforms DIAMOnD’s modular technique [30]. The ms RWR framework is capable of revealing components of the molecular mechanisms of psychiatric conditions. Our technique was effective in recapitulating accurate gene-gene interactions in metabolically distinct pathways, which offers insight to the relationship of SUDs and the underlying biological foundation. Given the outcome of an improved discriminative power with homological data, future experiments may leverage this technique for other metabolically-influenced diseases, for example cancer [106] or diabetes [107]. Hence, we have introduced this framework to emphasize genes within the context of SUDs and modify the performance of the RWR algorithm.

## Supporting information

S1 Table All raw and corrected post-hoc scores for Dunnet’s test comparisons (DOCX)

S1 FigGene Ontology closure example.The figure details a small example of how a GO tree is trimmed to only include ≤7 genes.(TIF)

S2 FigAmenability of ms RWR to noise and missing data.KW ANOVA results show no significance between any of the graphs, H-statistic = 3.05 and p-value = $8.02\;\times \;10^{-1}$.(TIF)

S3 FigComparison of performance of single species RWR with added noise compared to multi-species RWR.We conducted a comparison of single species RWR (ss RWR) with up to 30% added edges with our best performing algorithm, which was the multi-species RWR (ms RWR). (a) ss RWR with the addition of 10% edges. +10% ss RWR beat ms RWR in 4 of 13 pathways, which include the following: hsa00040, hsa00232, hsa00290, hsa04950. In pathway hsa04950, +10% ss RWR, *dp* = 4.42, edged ms RWR, *dp* = 4.23 (b) ss RWR with the addition of 20% edges. +20% ss RWR beat ms RWR in 4 out of 13 pathways, which include the following: hsa00040, hsa00232, hsa00290, hsa04950. In pathway hsa04950, +20% ss RWR, *dp* = 4.43, edged ms RWR, *dp* = 4.23 (c) ss RWR with the addition of 30% edges compared to ms RWR. +30% ss RWR beat ms RWR in 4 out of 13 pathways, which include the following: hsa00040, hsa00232, hsa00290, hsa04950. In pathway hsa04950, +30% ss RWR, *dp* = 4.45, edged ms RWR, *dp* = 4.23.(TIF)

S4 FigComparison of performance of single species RWR with missing data compared to multi-species RWR.We conducted a comparison of single species RWR (ss RWR) with up to 30% missing edges with our best performing algorithm, which was the multi-species RWR (ms RWR). (a) ss RWR with 10% missing edges. −10% ss RWR beat ms RWR in 3 of 13 pathways, which include the following: hsa00040, and hsa00232, and hsa00290. (b) ss RWR with 20% missing edges. −20% ss RWR beat ms RWR in 4 out of 13 pathways, which include the following: hsa00232, hsa00290, hsa00920, and hsa04950. For pathway hsa00040, ms RWR, *dp* = 2.97 edged −20% ss RWR, *dp* = 2.86. For pathway hsa00920, −20% ss RWR, *dp* = 7.40, edged ms RWR, *dp* = 7.05. For pathway hsa04950, −20% ss RWR, *dp* = 4.27, edged ms RWR, *dp* = 4.23. (c) ss RWR with 30% missing edges. −30% ss RWR beat ms RWR in 4 out of 13 pathways, which include the following: hsa00232, hsa00290, hsa00920, and hsa04950.(TIF)

S5 FigComparison of performance of multi-species RWR with added noise compared to single species RWR.We conducted a comparison of multi-species RWR (ms RWR) with up to 30% added edges with our best performing algorithm, which was the single species RWR (ss RWR). (a) ms RWR with the addition of 10% edged. +10% ms RWR beat ss RWR in 10 of 13 pathways, which include the following: hsa00140, hsa00563, hsa00670, hsa00920, hsa03020, hsa03022, hsa03430, hsa03450, hsa04130, and hsa04950. For pathway hsa04950, +10% ms RWR edged, *dp* = 4.40, ss RWR, *dp* = 4.37. (b) ms RWR with the addition of 20% edges. +20% ms RWR beat ss RWR in 10 out of 13 pathways, which include the following: hsa00140, hsa00563, hsa00670, hsa00920, hsa03020, hsa03022, hsa03430, hsa03450, hsa04130, and hsa04950. For pathway hsa04950, +20% ms RWR, *dp* = 4.43, edges ss RWR, *dp* = 4.37. (c) ms RWR with the addition of 30% edged compared to ss RWR. +30% ms RWR beat ss RWR in 10 out of 13 pathways, which include the following: hsa00140, hsa00563, hsa00670, hsa00920, hsa03020, hsa03022, hsa03430, hsa03450, hsa04130, and hsa04950. For hsa04950, ms RWR edged ss RWR with a discriminative power of 4.45, compared to 4.37 for ss RWR.(TIF)

S6 FigComparison of performance of multi-species RWR with missing data to single species RWR.We conducted a comparison of multi-species RWR (ms RWR) with up to 30% missing data with our best performing algorithm, which was the single species RWR (ss RWR) (a) ms RWR with 10% missing edges compared to ss RWR. −10% ms RWR beat ss RWR in the following 9 of 13 pathways: hsa00140, hsa00563, hsa00670, hsa00920, hsa03020, hsa03022, hsa03430, hsa03450, and hsa04130. For pathway hsa04950, ss RWR, *dp* = 4.37, edged ms RWR, *dp* = 4.31. (b) ms RWR with 20% missing edges compared to ss RWR. −20% ms RWR beat ss RWR in the following 9 out of 13 pathways: hsa00140, hsa00563, hsa00670, hsa00920, hsa03020, hsa03022, hsa03430, hsa03450, and hsa04130. For pathway hsa00232, ss RWR, *dp* = 4.74, edged −20% ms RWR, *dp* = 4.73. For pathway hsa03020, −20% ms RWR, *dp* = 4.47, edged ss RWR, *dp* = 4.45. For pathway hsa03450, −20% ms RWR, *dp* = 4.62, edged ss RWR, *dp* = 4.60. For pathway hsa04950, ss RWR, *dp* = 4.37, edged ms RWR, *dp* = 4.19. (c) ms RWR with 30% missing edges compared to ss RWR. −30% ms RWR beat ss RWR in the following 8 out of 13 pathways: hsa00140, hsa00232, hsa00563, hsa00670, hsa00920, hsa03430, hsa03450, and hsa04130. For pathway hsa03022, ss RWR, *dp* = 4.17 edged −30% ms RWR, 4.13. For pathway hsa03450, −30% ms RWR, *dp* = 4.62, edged ss RWR, *dp* = 4.60.(TIF)

S7 FigComparison of multi-species RWR data against single species semantic similarity measures.Probability value (*p*) annotation are as follows:*: $1.00\;\times \;10^{-2}<p\leq 5\;\times \;10^{-2}$, ****: $p\leq 1.00\;\times \;10^{-4}$ (a) Abbreviations are as follows: ms RWR is RWR with homology, jac is Jaccard, cos is cosine, res is Resnik, and lin is Lin. ms RWR outperformed all other semantic similarity measures in all 13 pathways. (b) Dunn’s test comparison of ms RWR with all semantic similarity measures. discriminative power was significantly higher than all other algorithms.(TIF)

S8 FigComparison of RWR with homologous species data against homologous semantic similarity measures.Probability value (*p*) annotation are as follows: : $1.00\;\times \;10^{-2}<p\leq 5\;\times \;10^{-2}$, ****: $p\leq 1.00\;\times \;10^{-4}$ (a) Abbreviations are as follows: ms RWR is RWR with homology, ms jac is multi-species Jaccard, ms cos is multi-species cosine, ms res is multi-species Resnik, and ms lin is multi-species Lin. ms RWR outperformed all other semantic similarity measures in all 13 pathways. (b) Dunn’s test comparison of ms RWR with all semantic similarity measures. discriminative power was significantly higher than all other algorithms.(TIF)

S9 FigComparison of multi-species and single species ontology lists.(a) We conducted a one-tailed Mann Whitney-U test of significance for missing values for each gene in each KEGG pathway. Missing values are genes which do not map to any GO annotation, and thereby cannot infer any semantic similarity score. We found that single species data had a higher amount of missing values whereas multi-species data had fewer, U-statistic = $1.19\;\times \;10^{2}$ and p-value = $3.84\;\times \;10^{-2}$. (b) We conducted a one-tailed independent samples t-test for log-transformed total ontology annotations for each gene in each KEGG pathway. Results were significant, t-statistic = 1.77 and p-value = $4.45\;\times \;10^{-2}$, for capturing more ontology annotations using homology clusters.(TIF)

S10 Fig Comparison of semantic similarity scoring with single species data and multi-species data. (a) Single species cosine (ss cos) outperformed multi-species cosine (ms cos) in all pathways, but was comparable in 2 pathways, hsa04950 and hsa03022. (b) Single species Jaccard (ss jac) outperformed multi-species Jaccard (ms jac) in all pathways, but was comparable in 2 pathways, hsa04950 and hsa03022. (c) Single species Lin (ss lin) outperformed multi-species Lin in 7 pathways, but performed worse in the following 3 pathways: hsa00232, hsa00563, hsa04130. They were comparable in 3 pathways hsa00290, hsa03020, and hsa00670. (d) Single species Resnik (ss res) outperformed multi-species Resnik (ms res) in all pathways.
(TIF)

## References

[1] Bough KJ, Pollock JD. Defining substance use disorders: the need for peripheral biomarkers. Trends Mol Med. 2018;24(2):109–20. doi: 10.1016/j.molmed.2017.12.009 29396146

[2] Hatoum AS, Colbert SMC, Johnson EC, Huggett SB, Deak JD, Pathak G, et al. Multivariate genome-wide association meta-analysis of over 1 million subjects identifies loci underlying multiple substance use disorders. Nat Ment Health. 2023;1(3):210–23. doi: 10.1038/s44220-023-00034-y 37250466 PMC10217792

[3] Abdellaoui A, Yengo L, Verweij KJH, Visscher PM. 15 years of GWAS discovery: realizing the promise. Am J Hum Genet. 2023;110(2):179–94. doi: 10.1016/j.ajhg.2022.12.011 36634672 PMC9943775

[4] Zitnik M, Li MM, Wells A, Glass K, Morselli Gysi D, Krishnan A, et al. Current and future directions in network biology. Bioinform Adv. 2024;4(1):vbae099. doi: 10.1093/bioadv/vbae099 39143982 PMC11321866

[5] Lee B, Zhang S, Poleksic A, Xie L. Heterogeneous multi-layered network model for omics data integration and analysis. Front Genet. 2020;10:1381. doi: 10.3389/fgene.2019.01381 32063919 PMC6997577

[6] Li MM, Huang K, Zitnik M. Graph representation learning in biomedicine and healthcare. Nat Biomed Eng. 2022;6(12):1353–69. doi: 10.1038/s41551-022-00942-x 36316368 PMC10699434

[7] Mahajan A, Spracklen CN, Zhang W, Ng MCY, Petty LE, Kitajima H, et al. Multi-ancestry genetic study of type 2 diabetes highlights the power of diverse populations for discovery and translation. Nat Genet. 2022;54(5):560–72. doi: 10.1038/s41588-022-01058-3 35551307 PMC9179018

[8] Park SL, Cheng I, Haiman CA. Genome-wide association studies of cancer in diverse populations. Cancer Epidemiol Biomarkers Prev. 2018;27(4):405–17. doi: 10.1158/1055-9965.EPI-17-0169 28637795 PMC5740019

[9] Uffelmann E, Huang QQ, Munung NS, de Vries J, Okada Y, Martin AR, et al. Genome-wide association studies. Nat Rev Methods Primers. 2021;1(1). doi: 10.1038/s43586-021-00056-9

[10] Santen GWE, Leitch HG, Cobben J. Gene-disease relationship evidence: a clinical perspective focusing on ultra-rare diseases. Hum Mutat. 2022;43(8):1082–8. doi: 10.1002/humu.24367 35266245 PMC9544306

[11] Brunham LR, Hayden MR. Hunting human disease genes: lessons from the past, challenges for the future. Hum Genet. 2013;132(6):603–17. doi: 10.1007/s00439-013-1286-3 23504071 PMC3654184

[12] Shu J, Li Y, Wang S, Xi B, Ma J. Disease gene prediction with privileged information and heteroscedastic dropout. Bioinformatics. 2021;37(Suppl_1):i410–7. doi: 10.1093/bioinformatics/btab310 34252957 PMC8275341

[13] Hernández-Lorenzo L, Hoffmann M, Scheibling E, List M, Matías-Guiu JA, Ayala JL. On the limits of graph neural networks for the early diagnosis of Alzheimer’s disease. Sci Rep. 2022;12(1):17632. doi: 10.1038/s41598-022-21491-y 36271229 PMC9587223

[14] Yamaguchi H, Hashimoto Y, Sugihara G, Miyata J, Murai T, Takahashi H, et al. Three-dimensional convolutional autoencoder extracts features of structural brain images with a “diagnostic label-free” approach: application to schizophrenia datasets. Front Neurosci. 2021;15:652987. doi: 10.3389/fnins.2021.652987 34305514 PMC8294943

[15] Andreassen OA, Hindley GFL, Frei O, Smeland OB. New insights from the last decade of research in psychiatric genetics: discoveries, challenges and clinical implications. World Psychiatry. 2023;22(1):4–24. doi: 10.1002/wps.21034 36640404 PMC9840515

[16] Alorf A, Khan MUG. Multi-label classification of Alzheimer’s disease stages from resting-state fMRI-based correlation connectivity data and deep learning. Comput Biol Med. 2022;151(Pt A):106240. doi: 10.1016/j.compbiomed.2022.106240 36423532

[17] Lynall M-E, Soskic B, Hayhurst J, Schwartzentruber J, Levey DF, Pathak GA, et al. Genetic variants associated with psychiatric disorders are enriched at epigenetically active sites in lymphoid cells. Nat Commun. 2022;13(1):6102. doi: 10.1038/s41467-022-33885-7 36243721 PMC9569335

[18] Gelernter J. Genetics of complex traits in psychiatry. Biol Psychiatry. 2015;77(1):36–42. doi: 10.1016/j.biopsych.2014.08.005 25444161 PMC4282183

[19] Palmer RHC, Johnson EC, Won H, Polimanti R, Kapoor M, Chitre A, et al. Integration of evidence across human and model organism studies: a meeting report. Genes Brain Behav. 2021;20(6):e12738. doi: 10.1111/gbb.12738 33893716 PMC8365690

[20] Sun J, Kranzler HR, Gelernter J, Bi J. A genome-wide association study of cocaine use disorder accounting for phenotypic heterogeneity and gene–environment interaction. J Psychiatry Neurosci. 2020;45(1):34–44. doi: 10.1503/jpn.180098 31490055 PMC6919916

[21] Zhou H, Rentsch CT, Cheng Z, Kember RL, Nunez YZ, Sherva RM, et al. Association of OPRM1 functional coding variant with opioid use disorder: a genome-wide association study. JAMA Psychiatry. 2020;77(10):1072–80. doi: 10.1001/jamapsychiatry.2020.1206 32492095 PMC7270886

[22] Reynolds T, Johnson EC, Huggett SB, Bubier JA, Palmer RHC, Agrawal A, et al. Interpretation of psychiatric genome-wide association studies with multispecies heterogeneous functional genomic data integration. Neuropsychopharmacology. 2021;46(1):86–97. doi: 10.1038/s41386-020-00795-5 32791514 PMC7688940

[23] Gerring ZF, Thorp JG, Treur JL, Verweij KJH, Derks EM. The genetic landscape of substance use disorders. Mol Psychiatry. 2024;29(11):3694–705. doi: 10.1038/s41380-024-02547-z 38811691 PMC11541208

[24] McCaw ZR, Gao J, Lin X, Gronsbell J. Synthetic surrogates improve power for genome-wide association studies of partially missing phenotypes in population biobanks. Nat Genet. 2024;56(7):1527–36. doi: 10.1038/s41588-024-01793-9 38872030 PMC11955959

[25] Nestler EJ. Is there a common molecular pathway for addiction?. Nat Neurosci. 2005;8(11):1445–9. doi: 10.1038/nn1578 16251986

[26] Zillich L, Poisel E, Frank J, Foo JC, Friske MM, Streit F, et al. Multi-omics signatures of alcohol use disorder in the dorsal and ventral striatum. Transl Psychiatry. 2022;12(1):190. doi: 10.1038/s41398-022-01959-1 35523767 PMC9076849

[27] Alabadla M, Sidi F, Ishak I, Ibrahim H, Affendey LS, Che Ani Z, et al. Systematic review of using machine learning in imputing missing values. IEEE Access. 2022;10:44483–502. doi: 10.1109/access.2022.3160841

[28] Zhang W, Chien J, Yong J, Kuang R. Network-based machine learning and graph theory algorithms for precision oncology. NPJ Precis Oncol. 2017;1(1):25. doi: 10.1038/s41698-017-0029-7 29872707 PMC5871915

[29] Lanciano T, Savino A, Porcu F, Cittaro D, Bonchi F, Provero P. Contrast subgraphs allow comparing homogeneous and heterogeneous networks derived from omics data. Gigascience. 2022;12:giad010. doi: 10.1093/gigascience/giad010 36852877 PMC9972522

[30] Gentili M, Martini L, Sponziello M, Becchetti L. Biological random walks: multi-omics integration for disease gene prioritization. Bioinformatics. 2022;38(17):4145–52. doi: 10.1093/bioinformatics/btac446 35792834

[31] Liu C-C, Tseng Y-T, Li W, Wu C-Y, Mayzus I, Rzhetsky A, et al. DiseaseConnect: a comprehensive web server for mechanism-based disease-disease connections. Nucleic Acids Res. 2014;42(Web Server issue):W137-46. doi: 10.1093/nar/gku412 24895436 PMC4086092

[32] Oerton E, Roberts I, Lewis PSH, Guilliams T, Bender A. Understanding and predicting disease relationships through similarity fusion. Bioinformatics. 2019;35(7):1213–20. doi: 10.1093/bioinformatics/bty754 30169824 PMC6449746

[33] Barabási A-L, Gulbahce N, Loscalzo J. Network medicine: a network-based approach to human disease. Nat Rev Genet. 2011;12(1):56–68. doi: 10.1038/nrg2918 21164525 PMC3140052

[34] Köhler S, Bauer S, Horn D, Robinson PN. Walking the interactome for prioritization of candidate disease genes. Am J Hum Genet. 2008;82(4):949–58. doi: 10.1016/j.ajhg.2008.02.013 18371930 PMC2427257

[35] Xiong Y, Guo M, Ruan L, Kong X, Tang C, Zhu Y, et al. Heterogeneous network embedding enabling accurate disease association predictions. BMC Med Genomics. 2019;12(Suppl 10):186. doi: 10.1186/s12920-019-0623-3 31865913 PMC6927100

[36] Ghiassian SD, Menche J, Barabási A-L. A DIseAse MOdule Detection (DIAMOnD) algorithm derived from a systematic analysis of connectivity patterns of disease proteins in the human interactome. PLoS Comput Biol. 2015;11(4):e1004120. doi: 10.1371/journal.pcbi.1004120 25853560 PMC4390154

[37] Petti M, Bizzarri D, Verrienti A, Falcone R, Farina L. Connectivity significance for disease gene prioritization in an expanding universe. IEEE/ACM Trans Comput Biol Bioinform. 2020;17(6):2155–61. doi: 10.1109/TCBB.2019.2938512 31484130

[38] Van Dongen S. Graph clustering via a discrete uncoupling process. SIAM J Matrix Anal & Appl. 2008;30(1):121–41. doi: 10.1137/040608635

[39] Navlakha S, Kingsford C. The power of protein interaction networks for associating genes with diseases. Bioinformatics. 2010;26(8):1057–63. doi: 10.1093/bioinformatics/btq076 20185403 PMC2853684

[40] Joodaki M, Ghadiri N, Maleki Z, Lotfi Shahreza M. A scalable random walk with restart on heterogeneous networks with Apache Spark for ranking disease-related genes through type-II fuzzy data fusion. J Biomed Inform. 2021;115:103688. doi: 10.1016/j.jbi.2021.103688 33545331

[41] Su C, Tong J, Zhu Y, Cui P, Wang F. Network embedding in biomedical data science. Brief Bioinform. 2020;21(1):182–97. doi: 10.1093/bib/bby117 30535359

[42] Valdeolivas A, Tichit L, Navarro C, Perrin S, Odelin G, Levy N, et al. Random walk with restart on multiplex and heterogeneous biological networks. Bioinformatics. 2019;35(3):497–505. doi: 10.1093/bioinformatics/bty637 30020411

[43] Wen Y, Song X, Yan B, Yang X, Wu L, Leng D, et al. Multi-dimensional data integration algorithm based on random walk with restart. BMC Bioinformatics. 2021;22(1):97. doi: 10.1186/s12859-021-04029-3 33639858 PMC7912853

[44] Duan G, Wu G, Chen X, Tian D, Li Z, Sun Y, et al. HGD: an integrated homologous gene database across multiple species. Nucleic Acids Res. 2023;51(D1):D994–1002. doi: 10.1093/nar/gkac970 36318261 PMC9825607

[45] Guo X, Liu R, Shriver CD, Hu H, Liebman MN. Assessing semantic similarity measures for the characterization of human regulatory pathways. Bioinformatics. 2006;22(8):967–73. doi: 10.1093/bioinformatics/btl042 16492685

[46] Hagberg A, Swart P, Chult S. Exploring network structure, dynamics, and function using NetworkX. Los Alamos, NM (United States): Los Alamos National Lab; 2008.

[47] McKinney W. Data structures for statistical computing in python. In: van der Walt S, Millman J, editors. Proceedings of the 9th Python in Science Conference; 2010. p. 56–61

[48] Virtanen P, Gommers R, Oliphant TE, Haberland M, Reddy T, Cournapeau D, et al. SciPy 1.0: fundamental algorithms for scientific computing in Python. Nat Methods. 2020;17(3):261–72. doi: 10.1038/s41592-019-0686-2 32015543 PMC7056644

[49] Kearney SK, Berger A, Baker E. Aon: a service to augment Alliance Genome Resource data with additional species. BMC Res Notes. 2023;16(1):297. doi: 10.1186/s13104-023-06577-8 37891644 PMC10604687

[50] Alliance of Genome Resources Consortium. Harmonizing model organism data in the Alliance of Genome Resources. Genetics. 2022;220(4):iyac022. doi: 10.1093/genetics/iyac022 35380658 PMC8982023

[51] Gene Ontology Consortium. The gene ontology resource: enriching a GOld mine. Nucleic Acids Res. 2021;49(D1):D325–34. doi: 10.1093/nar/gkaa1113 33290552 PMC7779012

[52] Oughtred R, Rust J, Chang C, Breitkreutz B-J, Stark C, Willems A, et al. The BioGRID database: a comprehensive biomedical resource of curated protein, genetic, and chemical interactions. Protein Sci. 2021;30(1):187–200. doi: 10.1002/pro.3978 33070389 PMC7737760

[53] Kanehisa M, Furumichi M, Tanabe M, Sato Y, Morishima K. KEGG: new perspectives on genomes, pathways, diseases and drugs. Nucleic Acids Res. 2017;45(D1):D353–61. doi: 10.1093/nar/gkw1092 27899662 PMC5210567

[54] Baker EJ, Jay JJ, Bubier JA, Langston MA, Chesler EJ. GeneWeaver: a web-based system for integrative functional genomics. Nucleic Acids Res. 2012;40(Database issue):D1067-76. doi: 10.1093/nar/gkr968 22080549 PMC3245070

[55] Sayers EW, Bolton EE, Brister JR, Canese K, Chan J, Comeau DC, et al. Database resources of the national center for biotechnology information. Nucleic Acids Res. 2022;50(D1):D20–6. doi: 10.1093/nar/gkab1112 34850941 PMC8728269

[56] Blake JA, Baldarelli R, Kadin JA, Richardson JE, Smith CL, Bult CJ, et al. Mouse Genome Database (MGD): knowledgebase for mouse-human comparative biology. Nucleic Acids Res. 2021;49(D1):D981–7. doi: 10.1093/nar/gkaa1083 33231642 PMC7779030

[57] Persson E, Sonnhammer ELL. InParanoiDB 9: ortholog groups for protein domains and full-length proteins. J Mol Biol. 2023;435(14):168001. doi: 10.1016/j.jmb.2023.168001 36764355

[58] Martin FJ, Amode MR, Aneja A, Austine-Orimoloye O, Azov AG, Barnes I, et al. Ensembl 2023. Nucleic Acids Res. 2023;51(D1):D933–41. doi: 10.1093/nar/gkac958 36318249 PMC9825606

[59] Thomas PD, Ebert D, Muruganujan A, Mushayahama T, Albou L-P, Mi H. PANTHER: making genome-scale phylogenetics accessible to all. Protein Sci. 2022;31(1):8–22. doi: 10.1002/pro.4218 34717010 PMC8740835

[60] Ashburner M, Ball CA, Blake JA, Botstein D, Butler H, Cherry JM, et al. Gene ontology: tool for the unification of biology. the gene ontology consortium. Nat Genet. 2000;25(1):25–9. doi: 10.1038/75556 10802651 PMC3037419

[61] Maglott D, Ostell J, Pruitt KD, Tatusova T. Entrez Gene: gene-centered information at NCBI. Nucleic Acids Res. 2007;35(Database issue):D26-31. doi: 10.1093/nar/gkl993 17148475 PMC1761442

[62] UniProt Consortium. UniProt: the universal protein knowledgebase in 2021. Nucleic Acids Res. 2021;49(D1):D480–9. doi: 10.1093/nar/gkaa1100 33237286 PMC7778908

[63] Castaneda EU, Baker EJ. KNeXT: a NetworkX-based topologically relevant KEGG parser. Front Genet. 2024;15:1292394. doi: 10.3389/fgene.2024.1292394 38415058 PMC10896898

[64] Alterovitz G, Xiang M, Mohan M, Ramoni MF. GO PaD: the gene ontology partition database. Nucleic Acids Res. 2007;35(Database issue):D322-7. doi: 10.1093/nar/gkl799 17098937 PMC1669720

[65] Piñero J, Ramírez-Anguita JM, Saüch-Pitarch J, Ronzano F, Centeno E, Sanz F, et al. The DisGeNET knowledge platform for disease genomics: 2019 update. Nucleic Acids Res. 2020;48(D1):D845–55. doi: 10.1093/nar/gkz1021 31680165 PMC7145631

[66] Barua JD, Omit SBS, Rana HK, Podder NK, Chowdhury UN, Rahman MH. Bioinformatics and system biological approaches for the identification of genetic risk factors in the progression of cardiovascular disease. Cardiovasc Ther. 2022;2022:9034996. doi: 10.1155/2022/9034996 36035865 PMC9381297

[67] Can T, Çamoundefinedlu O, Singh AK. Analysis of protein-protein interaction networks using random walks. In: Proceedings of the 5th International Workshop on Bioinformatics, 2005. p. 61–8.

[68] Wang JZ, Du Z, Payattakool R, Yu PS, Chen C-F. A new method to measure the semantic similarity of GO terms. Bioinformatics. 2007;23(10):1274–81. doi: 10.1093/bioinformatics/btm087 17344234

[69] Lin D. An information-theoretic definition of similarity. In: Proceedings of the Fifteenth International Conference on Machine Learning. ICML ’98. San Francisco, CA, USA: Morgan Kaufmann Publishers Inc.; 1998. p. 296–304

[70] Pesquita C, Faria D, Falcão AO, Lord P, Couto FM. Semantic similarity in biomedical ontologies. PLoS Comput Biol. 2009;5(7):e1000443. doi: 10.1371/journal.pcbi.1000443 19649320 PMC2712090

[71] Levandowsky M, Winter D. Distance between sets. Nature. 1971;234(5323):34–5.

[72] Resnik P. Using information content to evaluate semantic similarity in a taxonomy. In: Proceedings of the 14th International Joint Conference on Artificial Intelligence - Volume 1. IJCAI’95. San Francisco, CA, USA: Morgan Kaufmann Publishers Inc.; 1995. p. 448–453.

[73] Benabderrahmane S, Smail-Tabbone M, Poch O, Napoli A, Devignes M-D. IntelliGO: a new vector-based semantic similarity measure including annotation origin. BMC Bioinformatics. 2010;11:588. doi: 10.1186/1471-2105-11-588 21122125 PMC3098105

[74] Ehsani R, Drabløs F. TopoICSim: a new semantic similarity measure based on gene ontology. BMC Bioinformatics. 2016;17(1):296. doi: 10.1186/s12859-016-1160-0 27473391 PMC4966780

[75] Köhler S. Improved ontology-based similarity calculations using a study-wise annotation model. Database (Oxford). 2018;2018:bay026. doi: 10.1093/database/bay026 29688377 PMC5868182

[76] Cliff N. Dominance statistics: ordinal analyses to answer ordinal questions. Psychol Bull. 1993;114(3):494–509. doi: 10.1037/0033-2909.114.3.494

[77] Torchiano M. Effsize: efficient effect size computation. 2020. https://CRAN.R-project.org/package=effsize

[78] R Core Team. R: a language and environment for statistical computing; 2023. https://www.R-project.org/

[79] Hess MR, Kromrey JD. Robust confidence intervals for effect sizes: a comparative study of Cohen’sd and Cliff’s delta under non-normality and heterogeneous variances. In: Annual Meeting of the American Educational Research Association. vol. 1. Citeseer; 2004.

[80] Kane Meissel ESY. Using Cliff’s delta as a non-parametric effect size measure: an accessible web app and R tutorial. Practic Assessm Res Evaluat. 2024;29. doi: 10.7275/pare.1977

[81] Chen Y-A, Tripathi LP, Dessailly BH, Nyström-Persson J, Ahmad S, Mizuguchi K. Integrated pathway clusters with coherent biological themes for target prioritisation. PLoS One. 2014;9(6):e99030. doi: 10.1371/journal.pone.0099030 24918583 PMC4053319

[82] Chen Y, Xu D. Computational analyses of high-throughput protein-protein interaction data. Curr Protein Pept Sci. 2003;4(3):159–81. doi: 10.2174/1389203033487225 12769716

[83] Cheng Z, Peng Y, Wen J, Chen W, Pan W, Xu X, et al. Sex-specific metabolic signatures in methamphetamine addicts. Addict Biol. 2023;28(1):e13255. doi: 10.1111/adb.13255 36577725

[84] Cornelis MC, Flint A, Field AE, Kraft P, Han J, Rimm EB, et al. A genome-wide investigation of food addiction. Obesity (Silver Spring). 2016;24(6):1336–41. doi: 10.1002/oby.21476 27106561 PMC5038917

[85] Gaudet P, Dessimoz C. New York, NY: Springer; 2017. p. 189–205. 10.1007/978-1-4939-3743-1_14 27812944

[86] Zhao Y, Wang J, Chen J, Zhang X, Guo M, Yu G. A Literature review of gene function prediction by modeling gene ontology. Front Genet. 2020;11:400. doi: 10.3389/fgene.2020.00400 32391061 PMC7193026

[87] Szklarczyk D, Kirsch R, Koutrouli M, Nastou K, Mehryary F, Hachilif R, et al. The STRING database in 2023: protein-protein association networks and functional enrichment analyses for any sequenced genome of interest. Nucleic Acids Res. 2023;51(D1):D638–46. doi: 10.1093/nar/gkac1000 36370105 PMC9825434

[88] Wang W, Yang S, Li J. Drug target predictions based on heterogeneous graph inference. In: Pac Symp Biocomput, 2013. 53–64. 23424111 PMC3605000

[89] Liu W, Sun X, Peng L, Zhou L, Lin H, Jiang Y. RWRNET: a gene regulatory network inference algorithm using random walk with restart. Front Genet. 2020;11:591461. doi: 10.3389/fgene.2020.591461 33101398 PMC7545090

[90] Buzzao D, Castresana-Aguirre M, Guala D, Sonnhammer ELL. TOPAS, a network-based approach to detect disease modules in a top-down fashion. NAR Genom Bioinform. 2022;4(4):lqac093. doi: 10.1093/nargab/lqac093 36458021 PMC9706483

[91] Wang X-W, Qiao D, Cho MH, DeMeo DL, Silverman EK, Liu Y-Y. A statistical physics approach for disease module detection. Genome Res. 2022;32(10):1918–29. doi: 10.1101/gr.276690.122 36220609 PMC9712625

[92] Du Z, Li L, Chen C-F, Yu PS, Wang JZ. G-SESAME: web tools for GO-term-based gene similarity analysis and knowledge discovery. Nucleic Acids Res. 2009;37(Web Server issue):W345-9. doi: 10.1093/nar/gkp463 19491312 PMC2703883

[93] Jothi R, Mohanty SK, Ojha A. Functional grouping of similar genes using eigenanalysis on minimum spanning tree based neighborhood graph. Comput Biol Med. 2016;71:135–48. doi: 10.1016/j.compbiomed.2016.02.007 26945461

[94] Wang Y, Ma Y, Huang H, Wang B, Acharjya DP. A split–merge clustering algorithm based on the k-nearest neighbor graph. Inform Syst. 2023;111:102124. doi: 10.1016/j.is.2022.102124

[95] Arakelyan A, Nersisyan L. KEGGParser: parsing and editing KEGG pathway maps in Matlab. Bioinformatics. 2013;29(4):518–9. doi: 10.1093/bioinformatics/bts730 23292739

[96] Hosseini Z, Marashi S-A. Discovering missing reactions of metabolic networks by using gene co-expression data. Sci Rep. 2017;7:41774. doi: 10.1038/srep41774 28150713 PMC5288723

[97] Nersisyan L, Samsonyan R, Arakelyan A. CyKEGGParser: tailoring KEGG pathways to fit into systems biology analysis workflows. F1000Res. 2014;3:145. doi: 10.12688/f1000research.4410.2 25383185 PMC4215754

[98] Chen Y, Mao F, Li G, Xu Y. Genome-wide discovery of missing genes in biological pathways of prokaryotes. BMC Bioinformatics. 2011;12(Suppl 1):S1. doi: 10.1186/1471-2105-12-S1-S1 21342538 PMC3044263

[99] Benedict MN, Mundy MB, Henry CS, Chia N, Price ND. Likelihood-based gene annotations for gap filling and quality assessment in genome-scale metabolic models. PLoS Comput Biol. 2014;10(10):e1003882. doi: 10.1371/journal.pcbi.1003882 25329157 PMC4199484

[100] Green ML, Karp PD. A Bayesian method for identifying missing enzymes in predicted metabolic pathway databases. BMC Bioinformatics. 2004;5:76. doi: 10.1186/1471-2105-5-76 15189570 PMC446185

[101] King B, Farrah T, Richards MA, Mundy M, Simeonidis E, Price ND. ProbAnnoWeb and ProbAnnoPy: probabilistic annotation and gap-filling of metabolic reconstructions. Bioinformatics. 2018;34(9):1594–6. doi: 10.1093/bioinformatics/btx796 29267848

[102] Bernstein DB, Sulheim S, Almaas E, Segrè D. Addressing uncertainty in genome-scale metabolic model reconstruction and analysis. Genome Biol. 2021;22(1):64. doi: 10.1186/s13059-021-02289-z 33602294 PMC7890832

[103] Krumholz EW, Libourel IGL. Sequence-based network completion reveals the integrality of missing reactions in metabolic networks. J Biol Chem. 2015;290(31):19197–207. doi: 10.1074/jbc.M114.634121 26041773 PMC4521041

[104] Ponce-de-Leon M, Calle-Espinosa J, Peretó J, Montero F. Consistency analysis of genome-scale models of bacterial metabolism: a metamodel approach. PLoS One. 2015;10(12):e0143626. doi: 10.1371/journal.pone.0143626 26629901 PMC4668087

[105] Goddard ME, Kemper KE, MacLeod IM, Chamberlain AJ, Hayes BJ. Genetics of complex traits: prediction of phenotype, identification of causal polymorphisms and genetic architecture. Proc Biol Sci. 2016;283(1835):20160569. doi: 10.1098/rspb.2016.0569 27440663 PMC4971198

[106] Zaal EA, Berkers CR. The influence of metabolism on drug response in cancer. Front Oncol. 2018;8:500. doi: 10.3389/fonc.2018.00500 30456204 PMC6230982

[107] Zhang F, Chen X, Yang M, Shen X, Wang Y, Zhong D, et al. Metabolic impairments associated with type 2 diabetes mellitus and the potential effects of exercise therapy: an exploratory randomized trial based on untargeted metabolomics. PLoS One. 2024;19(3):e0300593. doi: 10.1371/journal.pone.0300593 38517904 PMC10959348

