# Supplementary material for: Influence of multi-species data on gene-disease associations in substance use disorder using random walk with restart models
Source: PLoS One. 2025 Jun 16;20(6):e0325201. doi: 10.1371/journal.pone.0325201 (PMC12169588; doi:10.1371/journal.pone.0325201)
Supplement: S1 Table — (DOCX) [file pone.0325201.s001.docx]

**S1 Table.** All raw and corrected post-hoc scores for Dunn's test comparisons.

|  |  | Uncorrected | | | |  | | Corrected* | | | | | |
| --- | --- | --- | --- | --- | --- | --- | --- | --- | --- | --- | --- | --- | --- |
|  |  | single species |  | multiple species |  | | single species | |  | | multiple species | |  |
| ss RWR | cos | $3.18{\times10}^{-2}$ |  | $1.11{\times10}^{-2}$ | |  | | $4.54{\times10}^{-2}$ | |  | | $1.58{\times10}^{-2}$ | |
|  | jac | $5.00{\times10}^{-2}$ |  | $1.91{\times10}^{-2}$ | |  | | $6.24{\times10}^{-2}$ | |  | | $2.38{\times10}^{-2}$ | |
|  | lin | $3.23{\times10}^{-8}$ |  | $2.15{\times10}^{-7}$ | |  | | $1.62{\times10}^{-7}$ | |  | | $1.00{\times10}^{-6}$ | |
|  | res | $2.31{\times10}^{-9}$ |  | $8.18{\times10}^{-12}$ | |  | | $2.31{\times10}^{-8}$ | |  | | $8.18{\times10}^{-11}$ | |
| ms RWR | cos | $1.48{\times10}^{-2}$ |  | $8.69{\times10}^{-3}$ | |  | | $2.11{\times10}^{-2}$ | |  | | $1.31{\times10}^{-2}$ | |
|  | jac | $2.37{\times10}^{-2}$ |  | $1.70{\times10}^{-2}$ | |  | | $2.69{\times10}^{-2}$ | |  | | $2.13{\times10}^{-2}$ | |
|  | lin | $1.03{\times10}^{-8}$ |  | $1.72{\times10}^{-7}$ | |  | | $5.16{\times10}^{-8}$ | |  | | $8.58{\times10}^{-7}$ | |
|  | res | $6.76{\times10}^{-10}$ |  | $6.12{\times10}^{-12}$ | |  | | $6.76{\times10}^{-9}$ | |  | | $6.12{\times10}^{-11}$ | |
| *Correction is through a Benjamini-Hochberg procedure | | | | | | | | | | | | | |
|  |  |  |  |  | |  | |  | |  | |  | |
