# Supplementary figures and images for: Influence of multi-species data on gene-disease associations in substance use disorder using random walk with restart models
Source: PLoS One. 2025 Jun 16;20(6):e0325201. doi: 10.1371/journal.pone.0325201 (PMC12169588; doi:10.1371/journal.pone.0325201)

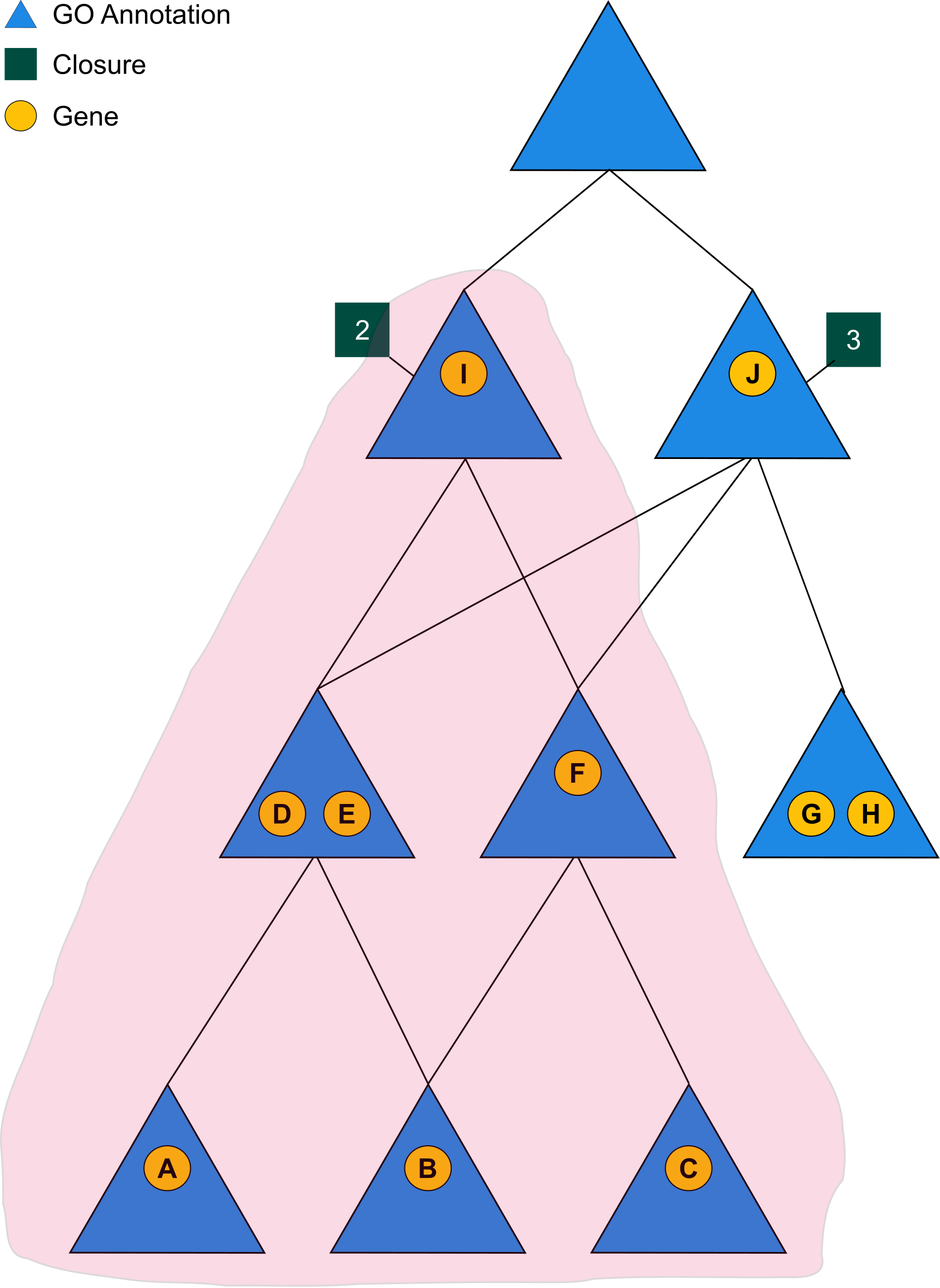

Supplement: S1 Fig — The figure details a small example of how a GO tree is trimmed to only include ≤7 genes. (TIF) [file pone.0325201.s002.tif]

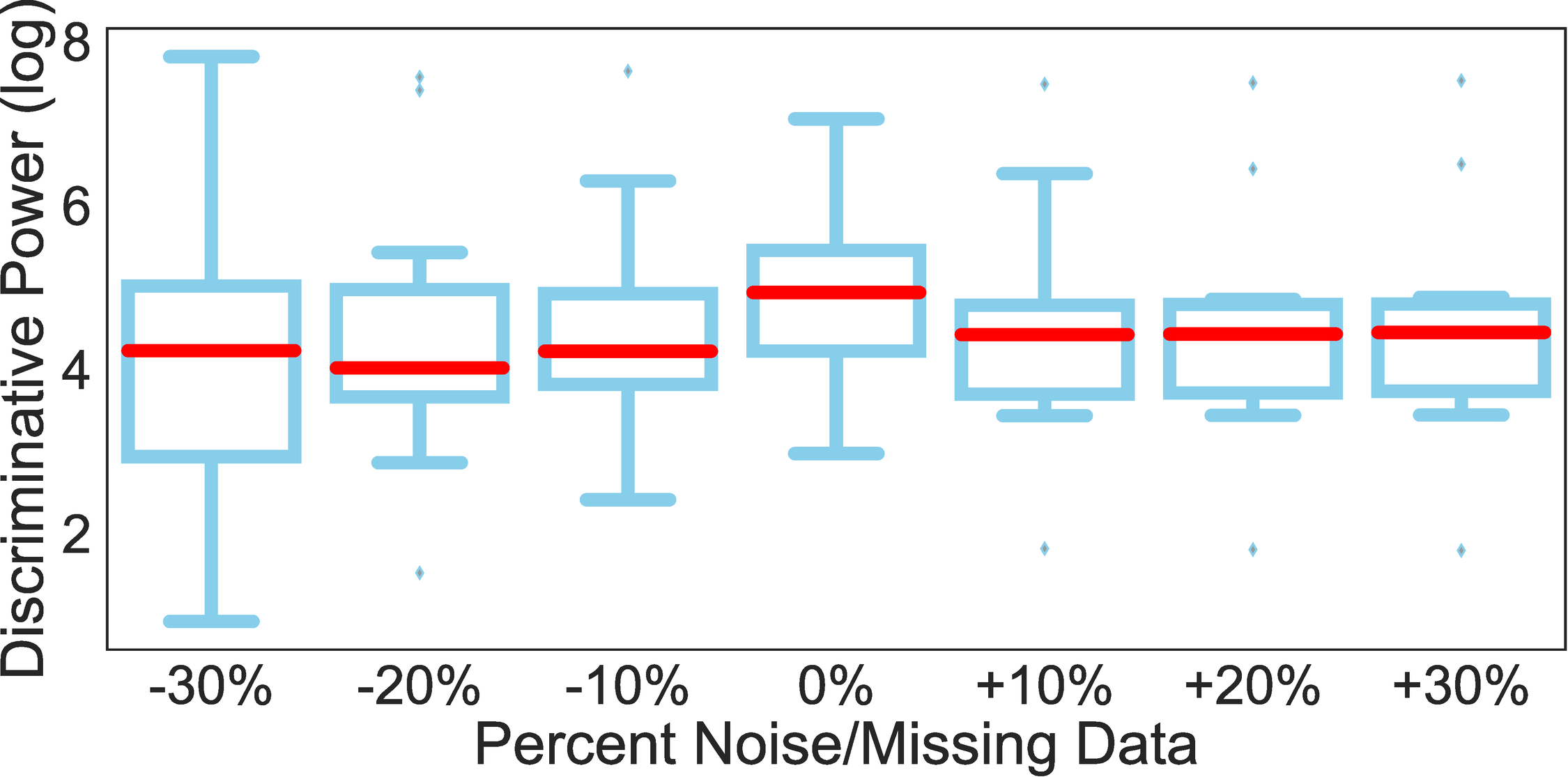

Supplement: S2 Fig — KW ANOVA results show no significance between any of the graphs, H-statistic = 3.05 and p-value = 8.02×10^−1^. (TIF) [file pone.0325201.s003.tif]

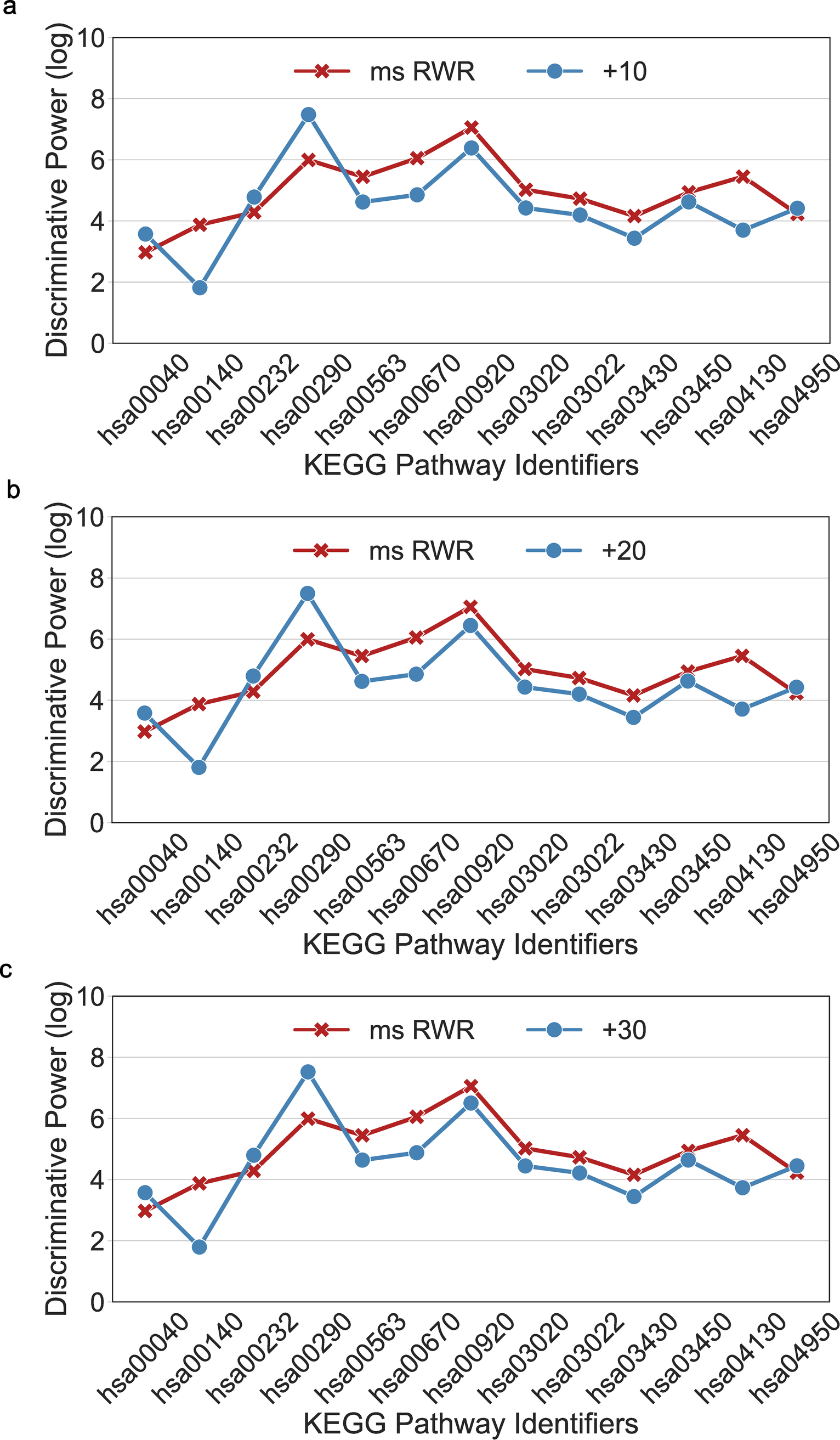

Supplement: S3 Fig — We conducted a comparison of single species RWR (ss RWR) with up to 30% added edges with our best performing algorithm, which was the multi-species RWR (ms RWR). (a) ss RWR with the addition of 10% edges. +10% ss RWR beat ms RWR in 4 of 13 pathways, which include the following: hsa00040, hsa00232, hsa00290, hsa04950. In pathway hsa04950, +10% ss RWR, dp = 4.42, edged ms RWR, dp = 4.23 (b) ss RWR with the addition of 20% edges. +20% ss RWR beat ms RWR in 4 out of 13 pathways, which include the following: hsa00040, hsa00232, hsa00290, hsa04950. In pathway hsa04950, +20% ss RWR, dp = 4.43, edged ms RWR, dp = 4.23 (c) ss RWR with the addition of 30% edges compared to ms RWR. +30% ss RWR beat ms RWR in 4 out of 13 pathways, which include the following: hsa00040, hsa00232, hsa00290, hsa04950. In pathway hsa04950, +30% ss RWR, dp = 4.45, edged ms RWR, dp = 4.23. (TIF) [file pone.0325201.s004.tif]

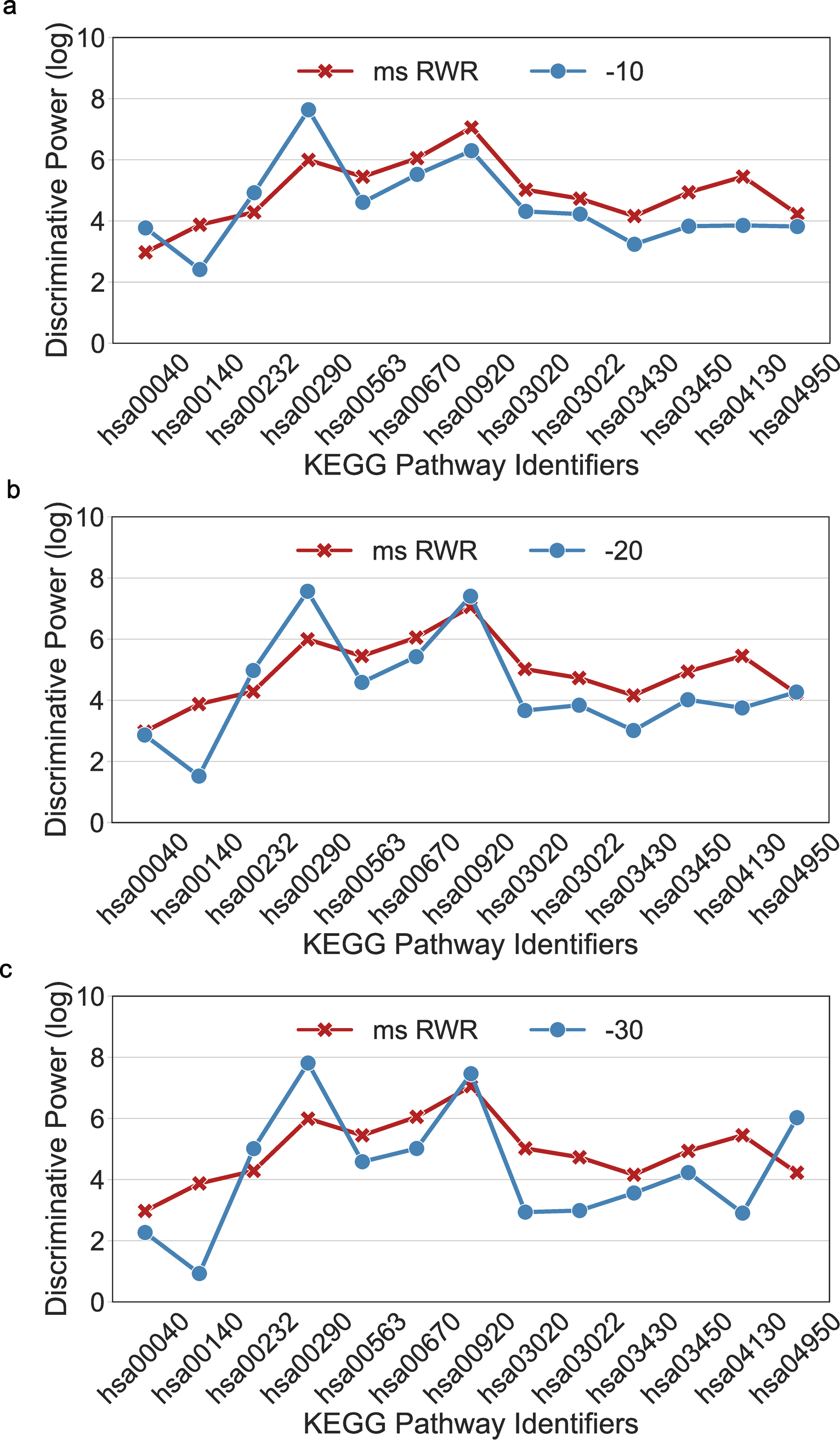

Supplement: S4 Fig — We conducted a comparison of single species RWR (ss RWR) with up to 30% missing edges with our best performing algorithm, which was the multi-species RWR (ms RWR). (a) ss RWR with 10% missing edges. −10% ss RWR beat ms RWR in 3 of 13 pathways, which include the following: hsa00040, and hsa00232, and hsa00290. (b) ss RWR with 20% missing edges. −20% ss RWR beat ms RWR in 4 out of 13 pathways, which include the following: hsa00232, hsa00290, hsa00920, and hsa04950. For pathway hsa00040, ms RWR, dp = 2.97 edged −20% ss RWR, dp = 2.86. For pathway hsa00920, −20% ss RWR, dp = 7.40, edged ms RWR, dp = 7.05. For pathway hsa04950, −20% ss RWR, dp = 4.27, edged ms RWR, dp = 4.23. (c) ss RWR with 30% missing edges. −30% ss RWR beat ms RWR in 4 out of 13 pathways, which include the following: hsa00232, hsa00290, hsa00920, and hsa04950. (TIF) [file pone.0325201.s005.tif]

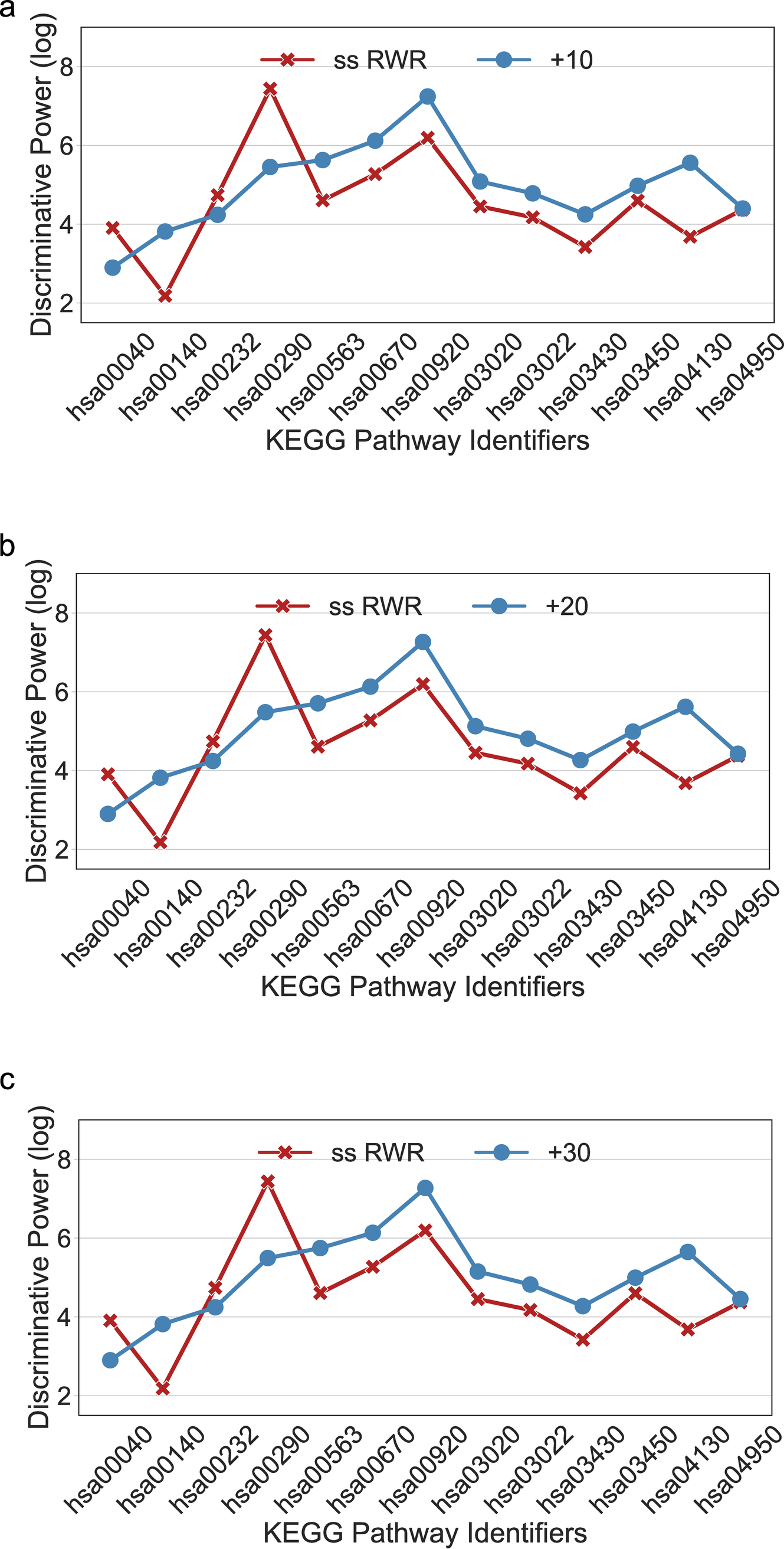

Supplement: S5 Fig — We conducted a comparison of multi-species RWR (ms RWR) with up to 30% added edges with our best performing algorithm, which was the single species RWR (ss RWR). (a) ms RWR with the addition of 10% edged. +10% ms RWR beat ss RWR in 10 of 13 pathways, which include the following: hsa00140, hsa00563, hsa00670, hsa00920, hsa03020, hsa03022, hsa03430, hsa03450, hsa04130, and hsa04950. For pathway hsa04950, +10% ms RWR edged, dp = 4.40, ss RWR, dp = 4.37. (b) ms RWR with the addition of 20% edges. +20% ms RWR beat ss RWR in 10 out of 13 pathways, which include the following: hsa00140, hsa00563, hsa00670, hsa00920, hsa03020, hsa03022, hsa03430, hsa03450, hsa04130, and hsa04950. For pathway hsa04950, +20% ms RWR, dp = 4.43, edges ss RWR, dp = 4.37. (c) ms RWR with the addition of 30% edged compared to ss RWR. +30% ms RWR beat ss RWR in 10 out of 13 pathways, which include the following: hsa00140, hsa00563, hsa00670, hsa00920, hsa03020, hsa03022, hsa03430, hsa03450, hsa04130, and hsa04950. For hsa04950, ms RWR edged ss RWR with a discriminative power of 4.45, compared to 4.37 for ss RWR. (TIF) [file pone.0325201.s006.tif]

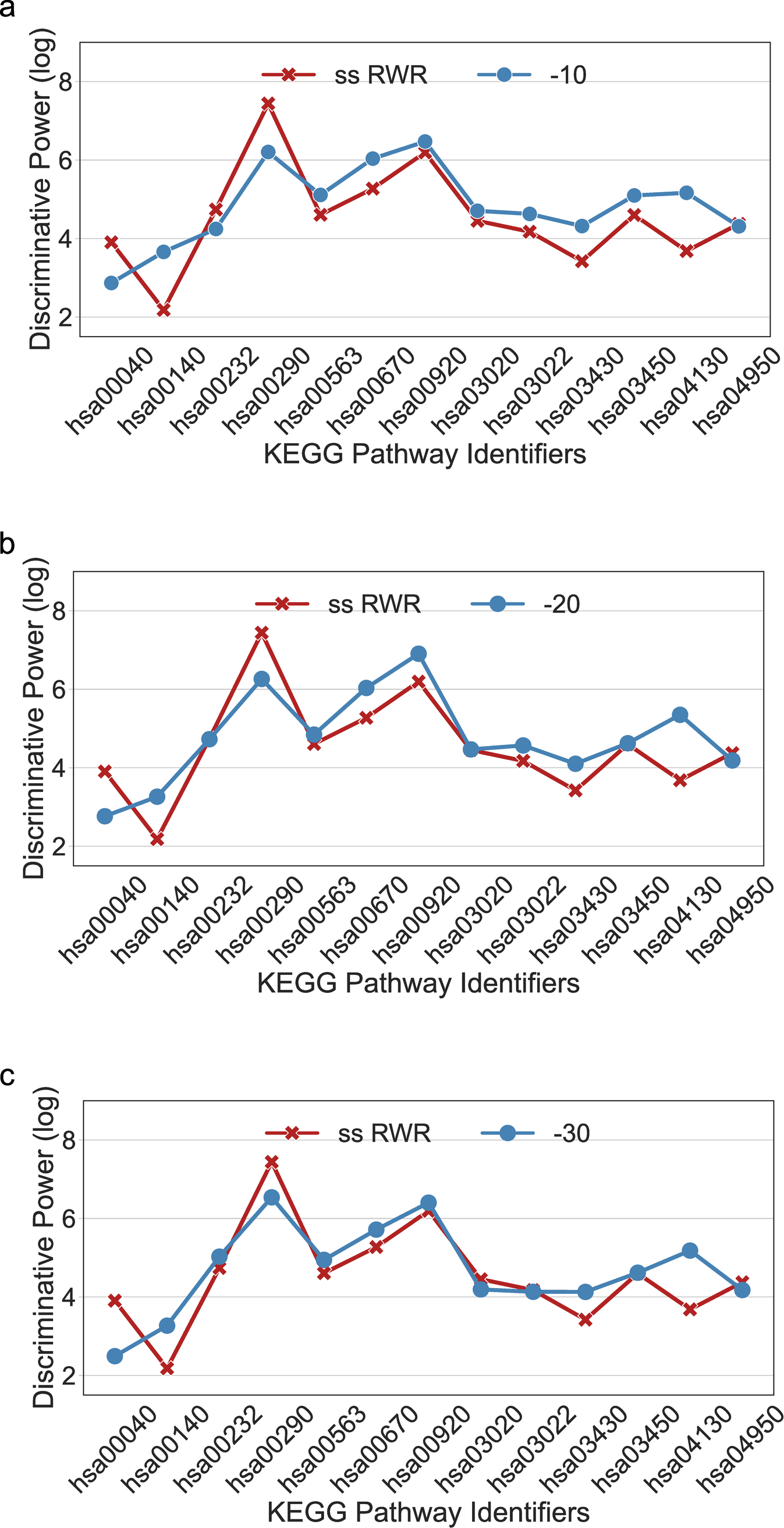

Supplement: S6 Fig — We conducted a comparison of multi-species RWR (ms RWR) with up to 30% missing data with our best performing algorithm, which was the single species RWR (ss RWR) (a) ms RWR with 10% missing edges compared to ss RWR. −10% ms RWR beat ss RWR in the following 9 of 13 pathways: hsa00140, hsa00563, hsa00670, hsa00920, hsa03020, hsa03022, hsa03430, hsa03450, and hsa04130. For pathway hsa04950, ss RWR, dp = 4.37, edged ms RWR, dp = 4.31. (b) ms RWR with 20% missing edges compared to ss RWR. −20% ms RWR beat ss RWR in the following 9 out of 13 pathways: hsa00140, hsa00563, hsa00670, hsa00920, hsa03020, hsa03022, hsa03430, hsa03450, and hsa04130. For pathway hsa00232, ss RWR, dp = 4.74, edged −20% ms RWR, dp = 4.73. For pathway hsa03020, −20% ms RWR, dp = 4.47, edged ss RWR, dp = 4.45. For pathway hsa03450, −20% ms RWR, dp = 4.62, edged ss RWR, dp = 4.60. For pathway hsa04950, ss RWR, dp = 4.37, edged ms RWR, dp = 4.19. (c) ms RWR with 30% missing edges compared to ss RWR. −30% ms RWR beat ss RWR in the following 8 out of 13 pathways: hsa00140, hsa00232, hsa00563, hsa00670, hsa00920, hsa03430, hsa03450, and hsa04130. For pathway hsa03022, ss RWR, dp = 4.17 edged −30% ms RWR, 4.13. For pathway hsa03450, −30% ms RWR, dp = 4.62, edged ss RWR, dp = 4.60. (TIF) [file pone.0325201.s007.tif]

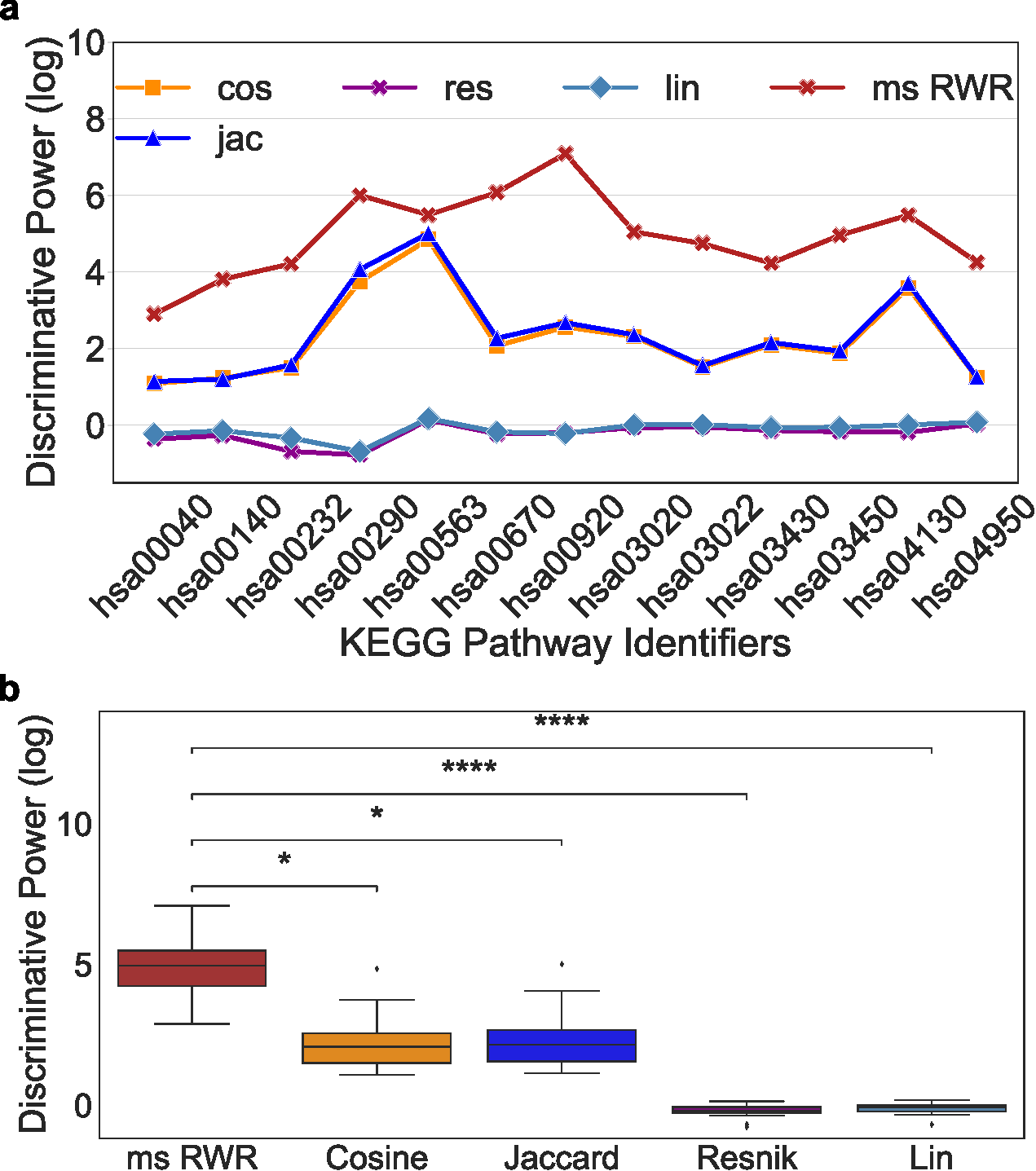

Supplement: S7 Fig — Probability value (p) annotation are as follows:*: 1.00×10^−2^<p≤5×10^−2^, ****: p≤1.00×10^−4^ (a) Abbreviations are as follows: ms RWR is RWR with homology, jac is Jaccard, cos is cosine, res is Resnik, and lin is Lin. ms RWR outperformed all other semantic similarity measures in all 13 pathways. (b) Dunn’s test comparison of ms RWR with all semantic similarity measures. discriminative power was significantly higher than all other algorithms. (TIF) [file pone.0325201.s008.tif]

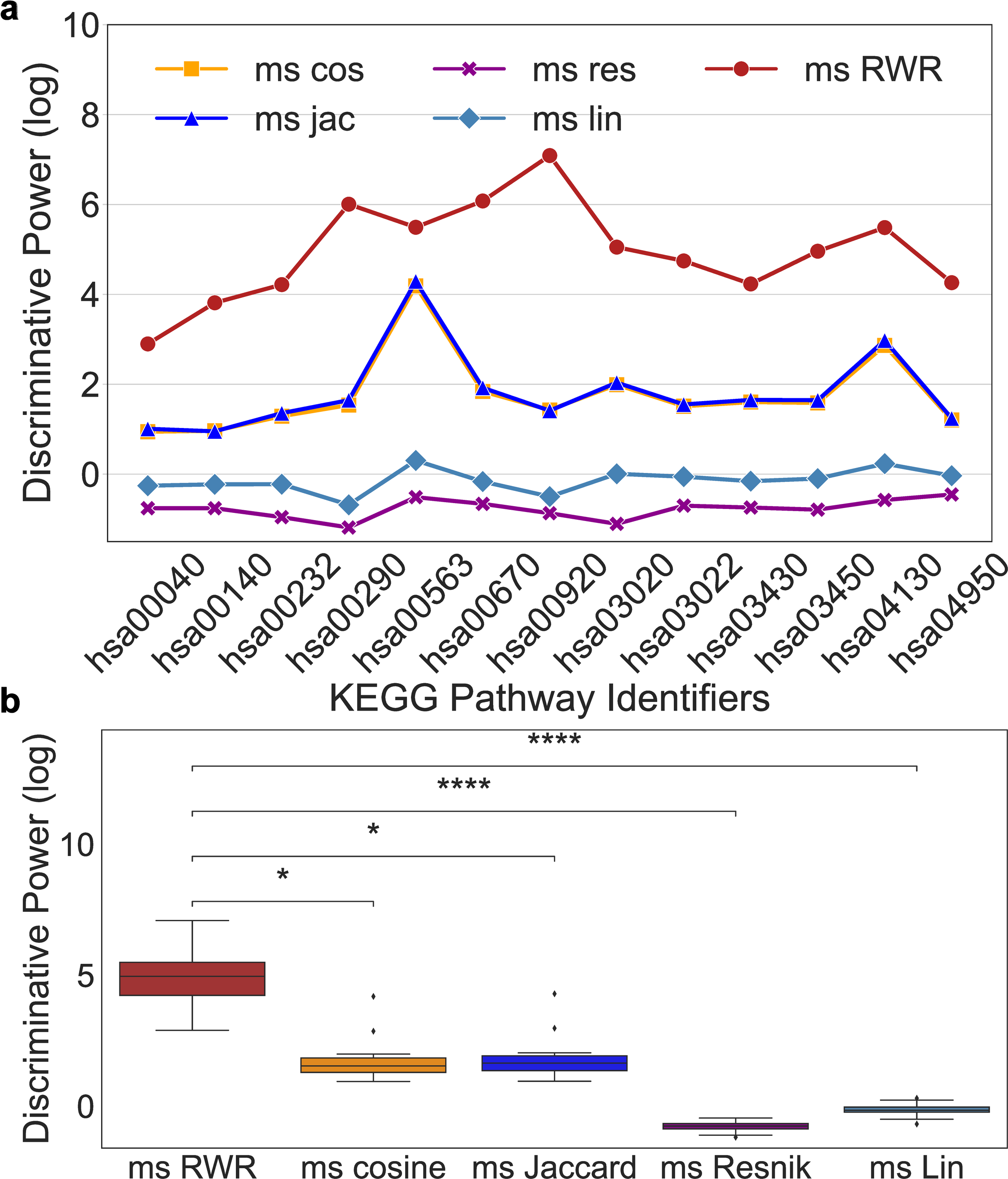

Supplement: S8 Fig — Probability value (p) annotation are as follows: : 1.00×10^−2^<p≤5×10^−2^, ****: p≤1.00×10^−4^ (a) Abbreviations are as follows: ms RWR is RWR with homology, ms jac is multi-species Jaccard, ms cos is multi-species cosine, ms res is multi-species Resnik, and ms lin is multi-species Lin. ms RWR outperformed all other semantic similarity measures in all 13 pathways. (b) Dunn’s test comparison of ms RWR with all semantic similarity measures. discriminative power was significantly higher than all other algorithms. (TIF) [file pone.0325201.s009.tif]

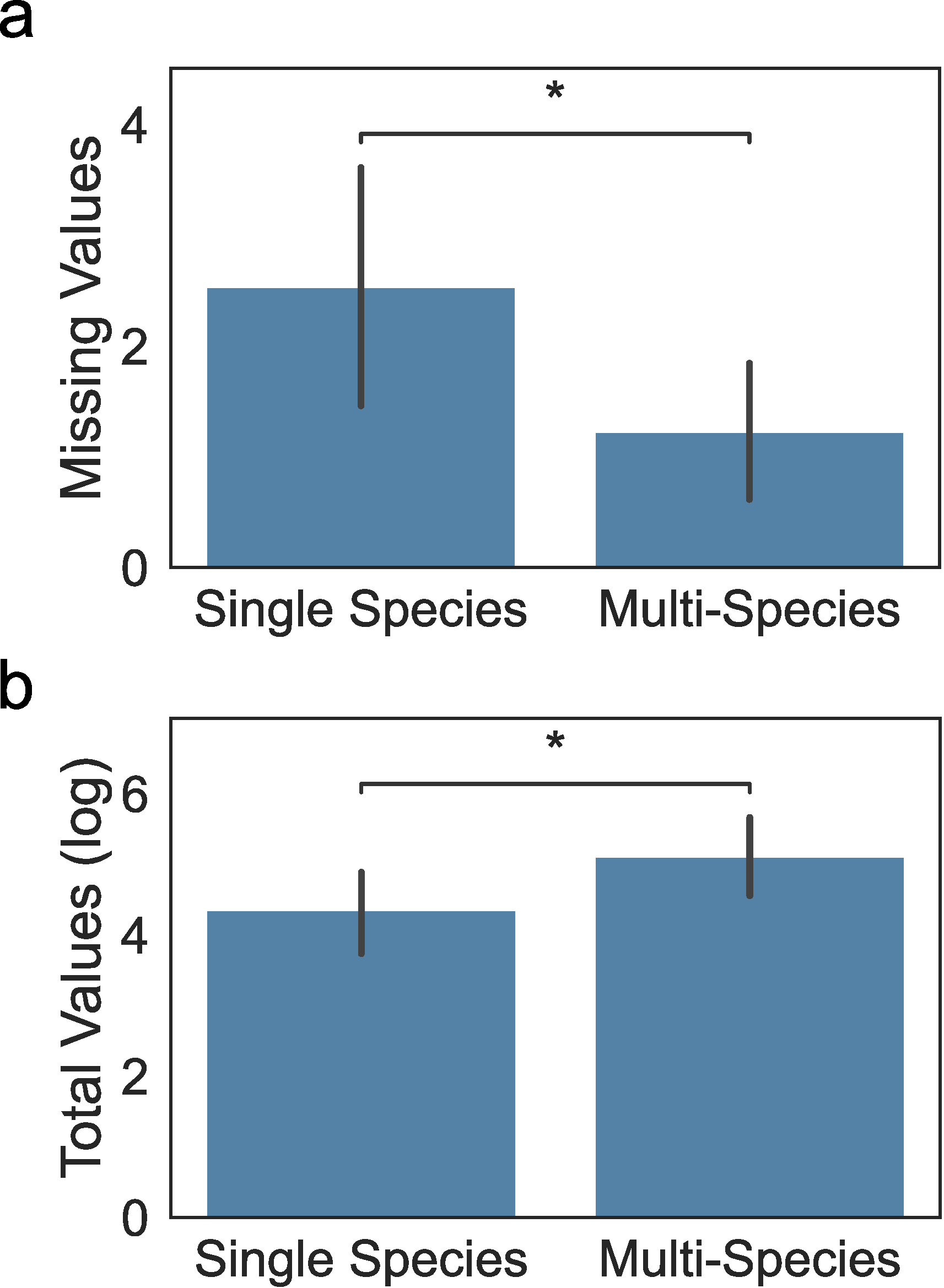

Supplement: S9 Fig — (a) We conducted a one-tailed Mann Whitney-U test of significance for missing values for each gene in each KEGG pathway. Missing values are genes which do not map to any GO annotation, and thereby cannot infer any semantic similarity score. We found that single species data had a higher amount of missing values whereas multi-species data had fewer, U-statistic = 1.19×10^2^ and p-value = 3.84×10^−2^. (b) We conducted a one-tailed independent samples t-test for log-transformed total ontology annotations for each gene in each KEGG pathway. Results were significant, t-statistic = 1.77 and p-value = 4.45×10^−2^, for capturing more ontology annotations using homology clusters. (TIF) [file pone.0325201.s010.tif]

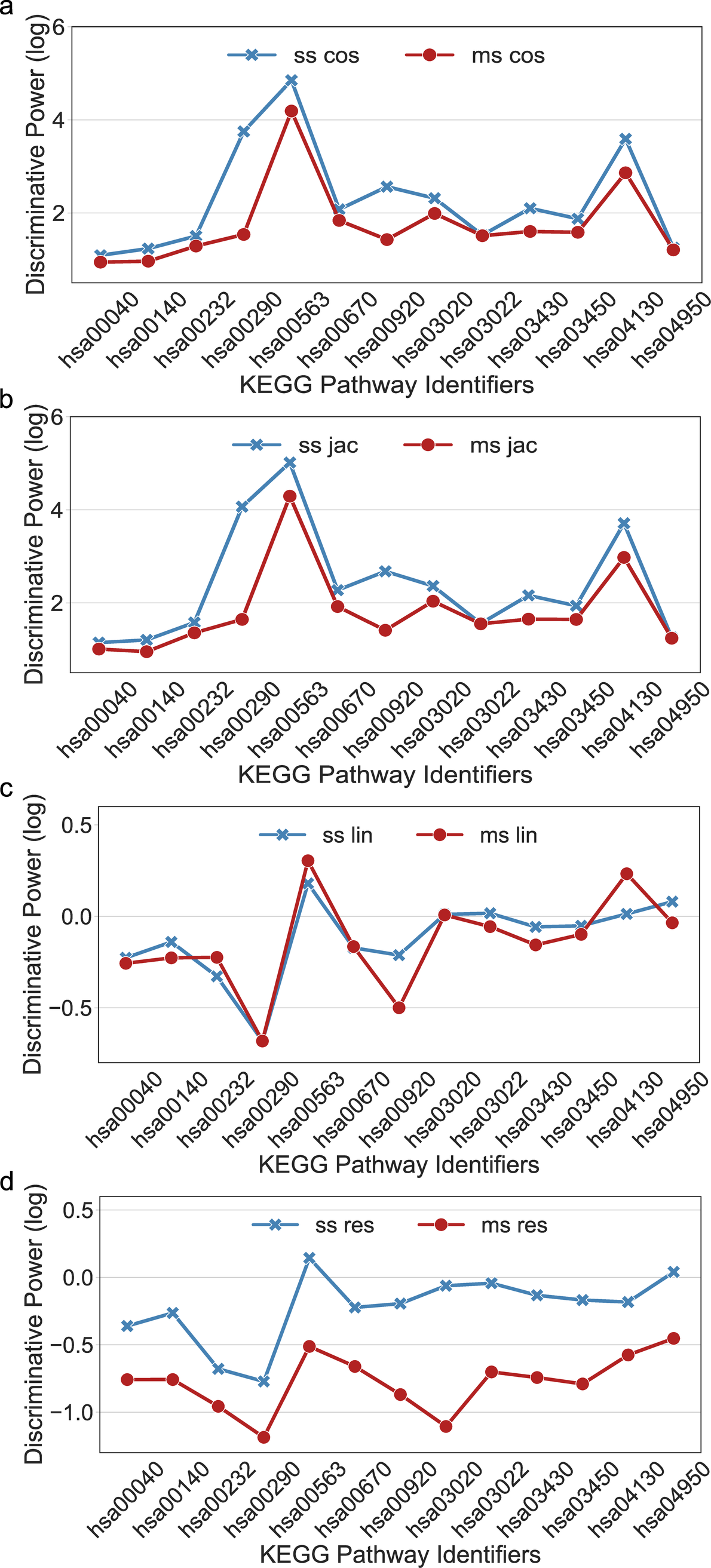

Supplement: S10 Fig — (a) Single species cosine (ss cos) outperformed multi-species cosine (ms cos) in all pathways, but was comparable in 2 pathways, hsa04950 and hsa03022. (b) Single species Jaccard (ss jac) outperformed multi-species Jaccard (ms jac) in all pathways, but was comparable in 2 pathways, hsa04950 and hsa03022. (c) Single species Lin (ss lin) outperformed multi-species Lin in 7 pathways, but performed worse in the following 3 pathways: hsa00232, hsa00563, hsa04130. They were comparable in 3 pathways hsa00290, hsa03020, and hsa00670. (d) Single species Resnik (ss res) outperformed multi-species Resnik (ms res) in all pathways. (TIF) [file pone.0325201.s011.tif]
